# Molecular Detection of 10 of the Most Unwanted Alien Forest Pathogens in Canada Using Real-Time PCR

**DOI:** 10.1371/journal.pone.0134265

**Published:** 2015-08-14

**Authors:** Josyanne Lamarche, Amélie Potvin, Gervais Pelletier, Don Stewart, Nicolas Feau, Dario I. O. Alayon, Angela L. Dale, Aaron Coelho, Adnan Uzunovic, Guillaume J. Bilodeau, Stephan C. Brière, Richard C. Hamelin, Philippe Tanguay

**Affiliations:** 1 Natural Resources Canada, Canadian Forest Service, Laurentian Forestry Centre, Québec, QC, Canada; 2 Department of Forest and Conservation Sciences, Faculty of Forestry, University of British Columbia, Vancouver, BC, Canada; 3 FPInnovations, Vancouver, BC, Canada; 4 Canadian Food Inspection Agency, Pathogen Identification Research Laboratory (PIRL), Ottawa, ON, Canada; 5 Canadian Food Inspection Agency, Pathogen Pathology Laboratory (PPL), Ottawa, ON, Canada; Naval Research Laboratory, UNITED STATES

## Abstract

Invasive alien tree pathogens can cause significant economic losses as well as large-scale damage to natural ecosystems. Early detection to prevent their establishment and spread is an important approach used by several national plant protection organizations (NPPOs). Molecular detection tools targeting 10 of the most unwanted alien forest pathogens in Canada were developed as part of the TAIGA project (http://taigaforesthealth.com/). Forest pathogens were selected following an independent prioritization. Specific TaqMan real-time PCR detection assays were designed to function under homogeneous conditions so that they may be used in 96- or 384-well plate format arrays for high-throughput testing of large numbers of samples against multiple targets. Assays were validated for 1) specificity, 2) sensitivity, 3) precision, and 4) robustness on environmental samples. All assays were highly specific when evaluated against a panel of pure cultures of target and phylogenetically closely-related species. Sensitivity, evaluated by assessing the limit of detection (with a threshold of 95% of positive samples), was found to be between one and ten target gene region copies. Precision or repeatability of each assay revealed a mean coefficient of variation of 3.4%. All assays successfully allowed detection of target pathogen on positive environmental samples, without any non-specific amplification. These molecular detection tools will allow for rapid and reliable detection of 10 of the most unwanted alien forest pathogens in Canada.

## Introduction

Invasive alien tree pathogens can cause significant economic losses as well as large-scale damage to natural ecosystems. Over the last century, Canada has experienced the dramatic consequences of introductions of alien forest pathogens. The pathogens responsible for white pine blister rust (*Cronartium ribicola* J.C. Fisch), beech bark disease (*Cryptococcus fagisuga* Lindinger), and Dutch elm disease (*Ophiostoma ulmi* (Buisman) Melin & Nannf. and *O*. *novo-ulmi* (Brasier)) were accidentally introduced into Canada and resulted in the death of millions of *Pinus strobus*, *P*. *monticola*, *P*. *albicaulis*, *Fagus grandifolia* Ehrh. and *Ulmus americana* L. trees throughout their distribution range. Despite public and institutional awareness of alien forest species, it is expected that their number and impact will keep increasing in the future [1, 2]. In order to implement quarantine and enforce mitigation measures following the introduction of exotic pathogens, national plant protection organizations (NPPOs) such as the Canadian Food and Inspection Agency (CFIA) need rapid, reliable, sensitive and accurate detection methods. The challenge for NPPOs is to be able to detect pathogens at their different life stages, including those that have the capacity to remain latent on asymptomatic tissues. Molecular detection using real-time PCR approaches allows for rapid, reliable and sensitive detection while simultaneously processing of large numbers of samples.

Real-time PCR has become the gold standard in pathogen detection in many fields, e.g. medicine, animal health, agriculture as well as forestry. It is sensitive enough to detect minute amounts of DNA from the target organism mixed with environmental material or host DNA, and the use of hydrolysis probes (TaqMan probes) offers an additional level of specificity, thereby enabling discrimination between closely related species with few polymorphic sites [3]. Real-time PCR also provides accurate quantification of target DNA in processed samples, which is directly proportional to the biomass of the targeted organism. For all these reasons, real-time PCR has been increasingly used to prevent and mitigate the introduction and dispersal of exotic and invasive plant pathogens.

So far, real-time PCR assays of forest pathogens have been mostly developed for single specific pathogens. However, real-time PCR using TaqMan probes offers the opportunity for multiplexing (multiple reactions in one tube [4]) and arraying (multiple reactions in separate tubes but on a single support), allowing for the simultaneous detection of a range of different pathogens in a large number of samples by performing a single real-time PCR run (e.g. [5–7]). Multiplexing usually requires extensive fine-tuning to avoid cross-reactivity and/or loss of sensitivity [8, 9]. This critical step can be circumvented by using arrays of assays operating under the same real-time PCR conditions, but running in as many tubes as there are assays included in the arrays.

The objective of the present study therefore was to develop and validate a set of sensitive, specific and precise real-time PCR assays for the rapid detection of 10 of the most unwanted alien forest fungal pathogens in Canada selected from a list of over 100 tree pathogens regulated by international, continental, and national phytosanitary organizations. Selection was based on the pathogen’s i) history of invasiveness, type and degree of damage/symptoms/pathogenicity, ii) host species and estimated economic impact, iii) dispersal pathways, establishment and adaptability, and iv) likelihood of establishment in Canada. Our goal was to develop assays that could be used either in simplex or in plate-based arrays.

## Materials and Methods

### Isolates selection

For each target pathogen, we built a panel of isolates encompassing multiple isolates of the target species and isolates from closely related species (sister species). Our selection of sister species was based on phylogenies found in recent scientific peer-reviewed studies [10–15] as well as on the advice of particular taxonomic group specialists. Cultures were obtained from collections (CBS, ATCC, as well as private ones) and stored in replicates at FPInnovations in Vancouver and the CFIA in Ottawa. When available, type cultures from referenced culture collections along with isolates provided by taxonomic authorities were preferred. To capture intraspecific genetic diversity, isolates from different hosts and different geographic origins were used when available. The list of isolates used to design the assays is presented in Table 1.

**Table 1:** Target and closely related species isolates used in this study.

Target species	Species	Collection number	Host	Origin	Source^a^
***Ceratocytis laricicola*, *C*. *polonica and C*. *fagacearum***	*Ambrosiella ferruginea*	CBS 408.68	-	WI, USA	CBS
	*A*. *ferruginea*	CBS 460.82	*Fagus sylvatica*	Germany	CBS
	*Ceratocystis adiposa*	UAMH 6973	*Picea* sp.	QC, Canada	UAMH
	*C*. *adiposa*	UAMH 6974	*Picea* sp.	QC, Canada	UAMH
	*C*. *albifundus*	CBS 128991	*Acacia mearnsii*	-	CBS
	*C*. *bhutanensis*	CMW 8242; CBS 112907	*Picea* sp.	Bhutan	M.J. Wingfield
	*C*. *cacaofunesta*	CBS 115169	*Theobroma cacao*	Eucador	CBS
	*C*. *cacaofunesta*	CBS 152.62	*Theobroma cacao*	Costa Rica	CBS
	*C*. *caryae*	CBS 114716	*Carya cordiformis*	IA, USA	CBS
	*C*. *caryae*	CBS 115168	*Carya ovata*	IA, USA	CBS
	*C*. *coerulescens*	C301	*Pinus banksiana*	MN, USA	T.C. Harrington
	*C*. *coerulescens*	C313; CBS 140.37	*Picea abies*	Germany	T. C. Harrington
	*C*. *coerulescens*	C693	-	Finland	T.C. Harrington
	*C*. *coerulescens*	C1423	*Larix kaempferi*	Japan	T. C. Harrington
	*C*. *coerulescens*	CPT9; CL1-2	-	-	C. Breuil
	*C*. *coerulescens*	CPT11; CL2-15	-	-	C. Breuil
	*C*. *coerulescens*	CPT12; CL2-25	NA	NA	C. Breuil
	*C*. *douglasii*	C324; CBS 556.97	*Pseudotsuga menziesii*	OR, USA	T. C. Harrington
	*C*. *douglasii*	C479	NA	NA	T. C. Harrington
	*C*. *eucalypti*	CMW 3254	*Eucalyptus sieberi*	Australia	M.J. Wingfield
	*C*. *fagacearum*	C460	*Quercus alba*	IA, USA	T.C. Harrington
	*C*. *fagacearum*	C465	*Quercus macrocarpa*	IA, USA	T.C. Harrington
	*C*. *fagacearum*	C505	*Quercus rubra*	MN, USA	T.C. Harrington
	*C*. *fagacearum*	C520	*Quercus alba*	MN, USA	T.C. Harrington
	*C*. *fagacearum*	C660	*Quercus macrocarpa*	IA, USA	T.C. Harrington
	*C*. *fagacearum*	CMW 2039	*Quercus* sp.	MN, USA	M.J. Wingfield
	*C*. *fujiensis*	CMW 1952	*Larix* sp.	Japan	M.J. Wingfield
	*C*. *fujiensis*	CMW 1965	*Larix* sp.	Japan	M.J. Wingfield
	*C*. *fujiensis*	CMW 1969	*Larix* sp.	Japan	M.J. Wingfield
	*C*. *laricicola*	C181; CBS 100207	*Larix* sp.	Scotland	T.C. Harrington
	*C*. *laricicola*	CMW 3212	*Larix* sp.	Scotland	M.J. Wingfield
	*C*. *moniliformis*	CBS 118243	*Pinus mercusii*	Indonesia	CBS
	*C*. *norvegica*	UAMH 11187	*Picea abies*	Norway	UAMH
	*C*. *norvegica*	UAMH 11190	*Picea abies*	Norway	UAMH
	*C*. *paradoxa*	UAMH 3314	-	-	UAMH
	*C*. *paradoxa*	UAMH 8784	*Cocos nucifera*	Jamaica	UAMH
	*C*. *pinicola*	C488; CMW 1311; CBS 100199	*Pinus sylvestris*	United Kingdom	T.C. Harrington
	*C*. *pinicola*	C490; CMW 1323; CBS 100200	*Pinus nigra*	United Kingdom	T.C. Harrington
	*C*. *pinicola*	C795; CBS 100201	*Pinus nigra*	United Kingdom	T.C. Harrington
	*C*. *platani*	CBS 127662	*Platanus orientalis*	Greece	CBS
	*C*. *platani*	CBS 129000	*Platanus* sp.	USA	CBS
	*C*. *polonica*	C320; CBS 228.83	*Picea abies*	Norway	T.C. Harrington
	*C*. *polonica*	CBS 133.38	-	Poland	CBS
	*C*. *polonica*	CPT2; NISK 93-208/10	*Picea abies*	Norway	C. Breuil
	*C*. *polonica*	CPT3; NISK 93-208/115; ATCC 201884	*Picea abies*	Norway	C. Breuil
	*C*. *polonica*	CPT4; CBS 100205; CMW 2224	*Picea abies*	Norway	C. Breuil
	*C*. *polonica*	CPT5; CBS 100206	*Picea jezoensis*	Japan	C. Breuil
	*C*. *polonica*	CPT6; CBS 119236	*Picea jezoensis*	Japan	C. Breuil
	*C*. *radicicola*	CMW 3186; CBS 114.47	*Phoenix sp*.	CA, USA	M.J. Wingfield
	*C*. *resinifera*	C50	*Picea engelmannii*	NM, USA	T. C. Harrington
	*C*. *resinifera*	Kasper	-	-	L. Bernier
	*C*. *resinifera*	PB 632	*Pinus banksiana*	NB, Canada	L. Bernier
	*C*. *rufipenni*	C608; CBS 100209	*Picea engelmannii*	BC, Canada	T.C. Harrington
	*C*. *rufipenni*	C613; 404/2	*Picea glauca*	BC, Canada	T.C. Harrington
	*C*. *smalleyi*	CBS 114724	*Carya cordiformis*	WI, USA	CBS
	*C*. *variospora*	CBS 114714	*Quercus robur*	IA, USA	CBS
	*C*. *variospora*	CBS 114715	*Quercus alba*	IA, USA	CBS
	*C*. *virescens*	CMW 11164	*Fagus americanum*	USA	M.J. Wingfield
	*Thielaviopsis australis*	CMW 2333	*Nothofagus cunninghamii*	Australia	M.J. Wingfield
	*T*. *australis*	CMW 2339	*Eucalyptus* sp.	Australia	M.J. Wingfield
	*T*. *basicola*	CMW 7624	*Cichorium* sp.	South Africa	M.J. Wingfield
	*T*. *basicola*	CMW 7625	*Cichorium* sp.	South Africa	M.J. Wingfield
***Fusarium circinatum***	*Fusarium anthophilum*	CBS 737.97; NRRL 13602	*Hippeastrum sp*.	Germany	CBS
	*F*. *bactridioides*	CBS 100057; NRRL 22201	-	AZ, USA	CBS
	*F*. *bulbicola*	CBS 220.76; NRRL 13618	*Nerine bowdenii*	Netherlands	CBS
	*F*. *circinatum*	CBS 405.97; NRRL 25331	*Pinus radiata*	CA, USA	CBS
	*F*. *circinatum*	FCC1045; DAOM 238088	*Pinus patula*	South Africa	K. Seifert
	*F*. *circinatum*	FCC2251; DAOM 238089	*Pinus patula*	Mexico	K. Seifert
	*F*. *circinatum*	FCC2253; DAOM 238090	*Pinus greggii*	Mexico	K. Seifert
	*F*. *circinatum*	FCC4869; DAOM 238091	*Pinus patula*	USA	K. Seifert
	*F*. *circinatum*	FCC4873; DAOM 238092	*Pinus patula*	USA	K. Seifert
	*F*. *circinatum*	FCC4874; DAOM 238093	*Pinus patula*	USA	K. Seifert
	*F*. *circinatum*	FCC4878; DAOM 238094	*Pinus patula*	USA	K. Seifert
	*F*. *circinatum*	FCC4880; DAOM 238095	*Pinus patula*	South Africa	K. Seifert
	*F*. *circinatum*	FCC4881; DAOM 238096	*Pinus patula*	Mexico	K. Seifert
	*F*. *circinatum*	FCC4885; DAOM 238097	*Pinus patula*	Mexico	K. Seifert
	*F*. *circinatum*	FCC4913; DAOM 238098	*Pinus leiophylla*	Mexico	K. Seifert
	*F*. *guttiforme*	CBS 409.97; NRRL 25295	*Ananas comosun*	Brazil	CBS
	*F*. *subglutinans*	CBS 215.76; NRRL 20844	*Zea mays*	Germany	CBS
	*F*. *subglutinans*	AAFC-Fcir-012	-	-	K. Seifert
	*F*. *sacchari*	AAFC-Fcir-014	-	-	K. Seifert
	*F*. *succisae*	AAFC-Fcir-001	-	-	K. Seifert
	*F*. *succisae*	AAFC-Fcir-013	-	-	K. Seifert
***Geosmithia morbida***	*Geosmithia argillacea*	CBS 128034	*Xylosandrus mutilatus/Vitus rotundifolia*	USA	CBS
	*G*. *argillacea*	CBS 128787	-	-	-
	*G*. *fassatiae*	CCF3334	*Quercus pubescens*	Czech Republic	Miroslav Kolarik
	*G*. *fassatiae*	CCF4331	*Pityophthorus* sp./ *Pinus sabiniana*	CA, USA	Miroslav Kolarik
	*G*. *fassatiae*	CCF4340	*Hylocurus hirtellus/Salix* sp.	CA, USA	Miroslav Kolarik
	*G*. *flava*	CCF3333	*Xiphydria* sp. /C*astanea sativa*	Czech Republic	Miroslav Kolarik
	*G*. *flava*	CCF4337	*Cerambycidae* sp./*Pseudotsuga douglasii*	CA, USA	Miroslav Kolarik
	*G*. *flava*	CCF4341	*Cryphalus pubescens/Sequoia serpervirens*	CA, USA	Miroslav Kolarik
	*G*. *langdonii*	CCF4326	*Phloeosinus cupressi/Cyperus groverianus*	CA, USA	Miroslav Kolarik
	*G*. *lavendula*	CCF4336	Bark beetle/*Pinus longaeva*	CA, USA	Miroslav Kolarik
	*G*. *morbida*	1223	*Pityophthorus juglandis/Juglans nigra*	UT, USA	Miroslav Kolarik
	*G*. *morbida*	1256	*Pityophthorus juglandis/Juglans nigra*	OR, USA	Miroslav Kolarik
	*G*. *morbida*	1259	*Pityophthorus juglandis/Juglans nigra*	OR, USA	Miroslav Kolarik
	*G*. *morbida*	1268	*Pityophthorus juglandis/Juglans nigra*	CA, USA	Miroslav Kolarik
	*G*. *morbida*	1271	*Pityophthorus juglandis/Juglans nigra*	CO, USA	Miroslav Kolarik
	*G*. *morbida*	1272	-	-	Miroslav Kolarik
	*G*. *morbida*	CCF3879; CBS 124664	*Pityophthorus juglandis/Juglans nigra*	CO, USA	Miroslav Kolarik
	*G*. *morbida*	CCF3880	*Pityophthorus juglandis/Juglans nigra*	AZ, USA	Miroslav Kolarik
	*G*. *morbida*	CCF3881; CBS 124663	*Pityophthorus juglandis/Juglans nigra*	CO, USA	Miroslav Kolarik
	*G*. *morbida*	Gm6	*Juglans* sp.	TN, USA	Ðenita Hadžiabdić Guerry
	*G*. *morbida*	Gm14	*Juglans* sp.	TN, USA	Ðenita Hadžiabdić Guerry
	*G*. *morbida*	Gm19	*Juglans* sp.	TN, USA	Ðenita Hadžiabdić Guerry
	*G*. *morbida*	Gm45	*Juglans* sp.	TN, USA	Ðenita Hadžiabdić Guerry
	*G*. *morbida*	U19	*Pityophthorus juglandis/Juglans hindsii*	CA, USA	Miroslav Kolarik
	*G*. *obscura*	CBS 121749	-	USA	CBS
	*G*. *pallida* s.s.	CCF4279	*Platypus janosoni/Gymnacranthera paniculata*	Papua New Guinea	Miroslav Kolarik
	*G*. *pallida sp*. *1*	MK1790	*Hypoborus ficus/Ficus carica*	Azerbaijan	Miroslav Kolarik
	*G*. *pallida sp*. *2*	CCF4315	*Scolytus rugulosus*, *Pseudothysanoes hopkinsi/Prunus* sp.	CA, USA	Miroslav Kolarik
	*G*. *pallida* sp. 5	CCF4271	*Scolytus multistriatus/Ulmus laevis*	Czech Republic	Miroslav Kolarik
	*G*. *pallida* sp. 23	CCF3639	*Scolytus rugulosus/Prunus armeniaca*	Turkey	Miroslav Kolarik
	*G*. *pallida* sp. MK1807	MK1807	Scolytid beetle/*Acacia smithii*	Australia	Miroslav Kolarik
	*G*. *putterillii*	CBS248.32	Soil	Netherlands	CBS
	*G*. *putterillii*	CCF3342	*Scolytus rugulosus/Prunus* sp.	Czech Republic	Miroslav Kolarik
	*G*. *putterillii*	CCF3442	*Liparthrum colchicum/Laurus nobilis*	France	Miroslav Kolarik
	*G*. *putterillii*	CCF4204	Bostrichid beetle/*Umbellularia californica*	CA, USA	Miroslav Kolarik
	*G*. *rufescens*	MK1821	*Cnesinus lecontei/Croton draco*	Costa Rica	Miroslav Kolarik
	*G*. sp. 8	CCF4277	*Scolytus intricatus/Quercus cerris*	Bulgaria	Miroslav Kolarik
	*G*. sp. 9	RJ0258	*Ips cembrae/Larix decidua*	Poland	Miroslav Kolarik
	*G*. sp. 10	CCF4282	*Hypoborus ficus/Ficus carica*	Turkey	Miroslav Kolarik
	*G*. sp. 11	CCF3555	*Scolytus intricatus/Quercus pubescens*	Hungary	Miroslav Kolarik
	*G*. sp. 12	CCF4320	*Hylesinus oregonus/Fraxinus* sp.	CO, USA	Miroslav Kolarik
	*G*. sp. 13	CCF3559	*Pteleobius vittatus/Ulmus minor*	Czech Republic	Miroslav Kolarik
	*G*. sp. 16	CCF4201	*Pityophthorus pityographus/Picea abies*	Poland	Miroslav Kolarik
	*G*. sp. 16-like	CCF4322	*Pityophthorus* sp., *Scolytus oregoni*, *Cryphalus pubescens/Pseudotsuga douglasii*	CO, USA	Miroslav Kolarik
	*G*. sp. 20	CCF3641	*Hypoborus ficus/Ficus carica*	France	Miroslav Kolarik
	*G*. sp. 20	CCF4303	*Hypoborus ficus/Ficus carica*	Syria	Miroslav Kolarik
	*G*. sp. 20	CCF4316	*Ips plastographus/Calocedrus decurrens*	CA, USA	Miroslav Kolarik
	*G*. sp. 20	MK764	*Phloetribus scarabeoides /Olea europea*	Syria	Miroslav Kolarik
	*G*. sp. 21	CCF4321	*Pityophthorus sp*.*/Pinus ponderosae*	CO, USA	Miroslav Kolarik
	*G*. sp. 21	CCF4334	*Phloesinus sp*.*/Cyperus occidentalis* var. *australis*	CA, USA	Miroslav Kolarik
	*G*. sp. 21	MK1665	*Hypoborus ficus/Ficus carica*	Spain	Miroslav Kolarik
	*G*. sp. 22	CCF3645	*Phloetribus scarabeoides scarabeoides/Olea europea*	Jordan	Miroslav Kolarik
	*G*. sp. 26	CCF4330	Nark beetle/*Pinus monophylla*	CA, USA	Miroslav Kolarik
	*G*. sp. 27	CCF4206	*Pityogenes bidentatus/Pinus silvestris*	Poland	Miroslav Kolarik
	*G*. sp. 29	CCF4199	*Cryphalus piceae* + *Pityophthorus pityographus/Abies alba*	Czech Republic	Miroslav Kolarik
	*G*. sp. 29	CCF4221	*Cryphalus piceae* + *Pityophthorus pityographus/Abies alba*	Czech Republic	Miroslav Kolarik
	*G*. sp. 30	CCF4220	*Pityogenes chalcographus/Picea abies*	Poland	Miroslav Kolarik
	*G*. sp. 31	CCF4328	Bark beetle/*Pinus muricata*	CA, USA	Miroslav Kolarik
	*G*. sp. 31	RJ21k	*Pityophthorus pityographus/Pinus sylivestris*	Poland	Miroslav Kolarik
	*G*. sp. 35	CCF4205	*Cryphalus piceae* + *Pityophthorus pityographus/Abies alba*	Czech Republic	Miroslav Kolarik
	*G*. sp. MK1820	CCF4292	*Cnesinus lecontei/Croton draco*	Costa Rica	Miroslav Kolarik
	*G*. sp. U410	CCF4324	*Pityophthorus sp*.*/Pinus sabineana*	CA, USA	Miroslav Kolarik
	*G*. sp. U410	CCF4332	*Pityophthorus sp*.*/Pinus sabineana*	CA, USA	Miroslav Kolarik
	*G*. *viridis*	CBS 252.87	-	Australia	CBS
***Gremmeniella abietina var*. *abietina* (EU race)**	*Gremmeniella abietina var*. *abietina* (EU race)	DAOM170389; ATCC34574; SN-2; 66.163/2	*Picea abies*	Norway	DAOM
	*G*. *abietina var*. *abietina* (EU race)	DAOM170402;SUS-9; 11-38D	*Pinus resinosa*	NY, USA	DAOM
	*G*. *abietina var*. *abietina* (EU race)	83–043	*Pinus resinosa*	QC, Canada	G. Laflamme
	*G*. *abietina var*. *abietina* (EU race)	DAOM170406; SW-2; ETH-7264	*Pinus cembrae*	Switzerland	DAOM
	*G*. *abietina var*. *abietina* (EU race)	DAOM170407; SW-3; ETH-7269	*Pinus cembrae*	Switzerland	DAOM
	*G*. *abietina var*. *abietina* (EU race)	DAOM170408; SW-4; ETH-7266	*Pinus cembrae*	Switzerland	DAOM
	*G*. *abietina var*. *abietina* (EU race)	Oulanka	*Pinus sylvestris*	Finland	A. Uotila & J. Kaitera
	*G*. *abietina var*. *abietina* (EU race)	Hedmark P.C.1.4	-	Norway	M. Vuorinen
	*G*. *abietina var*. *abietina* (EU race)	Kai 1.5	*Pinus sylvestris*	Finland	A. Uotila & J. Kaitera
	*G*. *abietina var*. *abietina* (EU race)	Hu 1.2X1.8	*Pinus sylvestris*	Finland	A. Uotila & J. Kaitera
	*G*. *abietina var*. *abietina* (EU race)	Toro 2.8X1-A1.8	*Pinus sylvestris*	Finland	A. Uotila & J. Kaitera
	*G*. *abietina var*. *abietina* (EU race)	Hedmark P.C.1.3	-	Norway	M. Vuorinen
	*G*. *abietina var*. *abietina* (EU race)	YN 1.4	*Pinus sylvestris*	Finland	A. Uotila & J. Kaitera
	*G*. *abietina var*. *abietina* (EU race)	Sup 1.2	*Pinus sylvestris*	Finland	A. Uotila & J. Kaitera
	*G*. *abietina var*. *abietina* (EU race)	Sup 1.4	*Pinus sylvestris*	Finland	A. Uotila & J. Kaitera
	*G*. *abietina var*. *abietina* (EU race)	SIU 1.3	*Pinus sylvestris*	Finland	A. Uotila & J. Kaitera
	*G*. *abietina var*. *abietina* (EU race)	Sup 1.6	*Pinus sylvestris*	Finland	A. Uotila & J. Kaitera
	*G*. *abietina var*. *abietina* (EU race)	Sup 1.8	*Pinus sylvestris*	Finland	A. Uotila & J. Kaitera
	*G*. *abietina var*. *abietina* (EU race)	Kai 1.7	*Pinus sylvestris*	Finland	A. Uotila & J. Kaitera
	*G*. *abietina var*. *abietina* (EU race)	KanKaan	*Pinus sylvestris*	Finland	A. Uotila & J. Kaitera
	*G*. *abietina var*. *abietina* (EU race)	Kai 1.8X1.8	*Pinus sylvestris*	Finland	A. Uotila & J. Kaitera
	*G*. *abietina var*. *abietina* (EU race)	Toro 2.6 X Sup 1.6	*Pinus sylvestris*	Finland	A. Uotila & J. Kaitera
	*G*. *abietina var*. *abietina* (EU race)	Muistomä	*Pinus sylvestris*	Finland	A. Uotila & J. Kaitera
	*G*. *abietina var*. *abietina* (EU race)	SUO 2.1	*Pinus sylvestris*	Finland	A. Uotila & J. Kaitera
	*G*. *abietina var*. *abietina* (EU race)	Sup1.1 X Sup 1.8	*Pinus sylvestris*	Finland	A. Uotila & J. Kaitera
	*G*. *abietina var*. *abietina* (EU race)	Orivesi	*Pinus sylvestris*	Finland	A. Uotila & J. Kaitera
	*G*. *abietina var*. *abietina* (EU race)	Kai 1.2	*Pinus sylvestris*	Finland	A. Uotila & J. Kaitera
	*G*. *abietina var*. *abietina* (EU race)	Toro 2.7	*Pinus sylvestris*	Finland	A. Uotila & J. Kaitera
	*G*. *abietina var*. *abietina* (EU race)	Pat 1.7	*Pinus sylvestris*	Finland	A. Uotila & J. Kaitera
	*G*. *abietina var*. *abietina* (EU race)	Sup 1.7	*Pinus sylvestris*	Finland	A. Uotila & J. Kaitera
	*G*. *abietina var*. *abietina* (EU race)	Sup 1.3	*Pinus sylvestris*	Finland	A. Uotila & J. Kaitera
	*G*. *abietina var*. *abietina* (EU race)	Viheriäis	*Pinus sylvestris*	Finland	A. Uotila & J. Kaitera
	*G*. *abietina var*. *abietina* (EU race)	Kai 1.8	*Pinus sylvestris*	Finland	A. Uotila & J. Kaitera
	*G*. *abietina var*. *abietina* (EU race)	Hyytiälä	*Pinus sylvestris*	Finland	A. Uotila & J. Kaitera
	*G*. *abietina var*. *abietina* (EU race)	Ahvenlampi	*Pinus sylvestris*	Finland	A. Uotila & J. Kaitera
	*G*. *abietina var*. *abietina* (EU race)	Kai 1.3	*Pinus sylvestris*	Finland	A. Uotila & J. Kaitera
	*G*. *abietina var*. *abietina* (EU race)	MH 1.3	*Pinus sylvestris*	Finland	A. Uotila & J. Kaitera
	*G*. *abietina var*. *abietina* (EU race)	Kai 1.6	*Pinus sylvestris*	Finland	A. Uotila & J. Kaitera
	*G*. *abietina var*. *abietina* (NA race)	DAOM170372; SC-39; HF-1	*Pinus resinosa*	-	DAOM
	*G*. *abietina var*. *abietina* (NA race)	DAOM170367; SC-25; WP-104	*Pinus strobus*	Canada	DAOM
	*G*. *abietina var*. *abietina* (Asian race)	Asia5.1	*Abies sachalinensis*	Japan	L. Bernier
	*G*. *abietina var*. *balsamea*	84–301	*Abies balsamea*	QC, Canada	G. Laflamme
	*G*. *laricina*	81–857	*Larix laricina*	QC, Canada	G. Laflamme
***Rosellinia necatrix***	*Rosellinia abscondita*	CBS 450.89	Driftwood	Switzerland	CBS
	*R*. *abscondita*	CBS 447.89	*Alnus incana*	Switzerland	CBS
	*R*. *aquila*	CBS 399.61	-	South Africa	CBS
	*R*. *britannica*	CBS 446.89	-	France	CBS
	*R*. *limoniispora*	CBS 382.86	*Triticum aestivum*	Switzerland	CBS
	*R*. *limoniispora*	CBS 283.64	-	-	CBS
	*R*. *necatrix*	CBS 349.36	*Malus sylvestris*	Argentina	CBS
	*R*. *necatrix*	CBS 267.30	*Narcissus pseudonarcissus*	Netherlands	CBS
	*R*. *nectrioides*	CBS 449.89	-	Sweden	CBS
	*R*. *thelena*	CBS 400.61	-	CA, USA	CBS
	*Entoleuca mammata*	CFL-2629	*Populus tremuloides*	QC, Canada	-
***Sclerotinia pseudotuberosa* (syn. *Ciboria batschiana*)**	*Botrytis cinerea*	CBS 131.28	*Linum usitatissimum*	Netherlands	CBS
	*B*. *cinerea*	DAOM 166439	-	-	DAOM
	*B*. *cinerea*	DAOM 192631	-	-	DAOM
	*B*. *cinerea*	DAOM 193576	-	-	DAOM
	*B*. *cinerea*	DAOM231368	-	-	DAOM
	*B*. *cinerea*	DAOM231371	-	-	DAOM
	*B*. *cinerea*	DAOM231372	-	-	DAOM
	*Ciboria americana*	CBS 117.24	*Castanea sativa*	-	CBS
	*Pycnopeziza sympodialis*	CBS 141.83	*Arctostaphylos uva-ursi*	Switzerland	CBS
	*P*. *sympodialis*	CBS 332.39	-	USA	CBS
	*Sclerotinia bulborum*	CBS 297.31	-	USA	CBS
	*S*. *minor*	CBS 339.39	*Lactuca sativa*	Italy	CBS
	*S*. *minor*	DAOM 191806	-	-	DAOM
	*S*. *pseudotuberosa*	CBS 312.37	*Quercus* sp.	Netherlands	CBS
	*S*. *pseudotuberosa*	CBS 327.75	*Quercus peduculata*	France	CBS
	*S*. *pseudotuberosa*	CBS 331.35	-	Italy	CBS
	*S*. *pseudotuberosa*	CBS 655.78	*Quercus robur*	Netherlands	CBS
	*S*. *sclerotiorum*	CBS 499.50	-	Netherlands	CBS
	*S*. *sclerotiorum*	DAOM 180751	-	-	DAOM
	*S*. *sclerotiorum*	DAOM 241671	-	-	DAOM
	*S*. *trifoliorum*	CBS 171.24	*Trifolium incarnatum*	-	CBS
***Phytophthora kernoviae* and *P*. *ramorum***	*Phytophthora boehmeriae*	CBS 100410	-	Australia	CBS
	*P*. *boehmeriae*	CBS 291.29; IMI180614	*Boehmeria nivea*	Taiwan	CBS
	*P*. *brassicae*	CBS 179.87	*Brassica oleraceae*	Netherlands	CBS
	*P*. *brassicae*	P10414; CBS113350	*Brassica oleraceae*	Netherlands	CBS
	*P*. *captiosa*	CBS 119107	*Eucalyptus saligna*	New Zealand	CBS
	*P*. *cryptogea*	CBS 113.19	*Lycopersicon esculentum*	Ireland	CBS
	*P*. *cryptogea*	CBS 418.71	*Gerbera* sp.	Netehrlands	CBS
	*P*. *cryptogea*	P1088; ATCC 46721; CBS 290.35; CBS 130866	*Aster* sp.	USA	CBS
	*P*. *drechsleri*	CBS 292.35	*Beta vulgaris* var. *altissima*	CA, USA	CBS
	*P*. *erythroseptica*	Br 664	-	-	G. J. Bilodeau
	*P*. *erythroseptica*	DAOM 233917	-	-	G. J. Bilodeau
	*P*. *fallax*	CBS 119109	*Eucalyptus delegatensis*	New Zealand	CBS
	*P*. *foliorum*	CBS 121665; ATCC MYA-3638; CMW 31064	Azalea	TN, USA	M. J. Wingfield
	*P*. *gallica*	CBS 117475	-	Germany	CBS
	*P*. *hibernalis*	1341320–3	-	CA, USA	G. J. Bilodeau
	*P*. *hibernalis*	P3822; ATCC 56353; CBS 114104; IMI1 34760	*Citrus sinensis*	Australia	CBS
	*P*. *insolita*	P6195; ATCC 56964; CBS 691.79; IMI 288805	-	Taiwan	CBS
	*P*. *kernoviae*	CBS 122049; CMW 31066; PD 06/3121107	*Rododendron sp*.	United Kingdom	CBS
	*P*. *kernoviae*	CBS 122208; CMW 31065; PD 0502010595	*Rhododendron ponticum*	United Kingdom	CBS
	*P*. *lateralis*	CBS 102608	-	CA, USA	G. J. Bilodeau
	*P*. *lateralis*	CBS 117106	*Chamaecyparis lawsoniana*	Netherlands	G. J. Bilodeau
	*P*. *lateralis*	CBS 168.42	*Chamaecyparis lawsoniana*	OR, USA	G. J. Bilodeau
	*P*. *lateralis*	Hansen 366	*Chamaecyparis lawsoniana*	USA	G. J. Bilodeau
	*P*. *lateralis*	Hansen 368	*Chamaecyparis lawsoniana*	USA	G. J. Bilodeau
	*P*. *morindae*	CBS 121982	*Morinda citrifolia*	HI, USA	CBS
	*P*. *porri*	CBS 114101	*Parthenium argentatum*	Australia	CBS
	*P*. *primulae*	CBS 114346	*Primula polyantha*	New Zealand	CBS
	*P*. *primulae*	P10333; CBS 620.97	*Primula acaulis*	Germany	CBS
	*P*. *quininea*	CBS 407.48	*Cinchona officinalis*	Peru	CBS
	*P*. *ramorum* (EU1)	03–0107	*Rhododendron* sp.	Canada	G. J. Bilodeau
	*P*. *ramorum* (NA1)	04–0002	*Camellia* sp.	Canada	G. J. Bilodeau
	*P*. *ramorum* (NA2)	04–0437	*Pyracantha koidzumii* "Victory"	Canada	G. J. Bilodeau
	*P*. *ramorum* (NA2)	10–3892	*Rhododendron* sp.	Canada	G. J. Bilodeau
	*P*. *ramorum* (EU1)	BBA 14-98-a; CBS 101550	*Rhododendron catawbienses*	Germany	G. J. Bilodeau
	*P*. *ramorum* (EU1)	BBA 9/95	*Rhododendron catawbienses*	Germany	G. J. Bilodeau
	*P*. *ramorum* (EU1)	CBS 101553	*Rhododendron catawbienses*	Germany	CBS
	*P*. *ramorum* (EU1)	P10301; CBS 101329	*Rhododendron* sp.	Netherlands	CBS
	*P*. *ramorum* (NA1)	Pr 52; CBS 110537	*Rhododendron* sp.	CA, USA	G. J. Bilodeau
	*P*. *ramorum* (NA2)	Pr1270626-1	*Peiris japonica*	CA, USA	G. J. Bilodeau
	*P*. *richardiae*	CBS 240.30	*Zantedeschia aethiopica*	USA	CBS
	*P*. *sp*. *"sansomea"*	CBS 117693	*Glycine max*	Ireland	CBS
	*P*. *sp*. *"sansomea"*	P3163; CBS117692	*Silene latifolia* subsp. *alba*	USA	CBS
	*P*. *syringae*	CBS 114107	*Prunus dulcis*	CA, USA	CBS
	*P*. *syringae*	P10330; CBS110161	*Rhododendron* sp.	Germany	CBS
	*P*. *trifolii*	CBS 117687	*Trofolium* sp.	MS, USA	CBS

^a^ CBS: The Centraalbureau voor Schimmelcultures collection; DAOM: Agriculture and Agri-Food Canada Fungal collection; UAMH: University of Alberta Microfungus Collection and Herbarium

### DNA extraction

For all isolates (except for *Ceratocystis* species), DNA was extracted using the Qiagen’s DNeasy Plant Mini Kit (Qiagen, Valencia, CA, USA) according to the manufacturer’s instructions. *Ceratocystis* DNA was extracted using a modified version of Zolan and Pukkila’s phenol/chloroform extraction protocol [16]. A small piece of mycelium was homogenized in 400 μl of extraction buffer (100mM Tris-HCl pH 9.5, 1.4 M NaCl, 20 mM EDTA, 2% CTAB, 1% PEG 8000, and 0.25% β-mercaptoethanol). Samples were then incubated at 65°C for 1h (vortexing every 15 minutes). Next, 400 μl of phenol:chloroform:isoamyl alcohol (25:24:1) were added to the homogenate, vortexed for 10 s and centrifuged at 13,000 x *g* for 10 min. Supernatant was mixed by inversion with 70 μl of 7.5M ammonium acetate and 600 μl of ice-cold isopropanol, incubated at -20°C for a minimum of 1h, and then centrifuged at 13,000 x *g* for 20 min (4°C). DNA was rinsed with 800 μl ice-cold 70% ethanol and centrifuged at 13,000 x *g* for 5 min (4°C). DNA was then incubated at 55°C to evaporate any remaining ethanol and re-suspended in 50 μl 10 mM Tris-HCl, pH 8.0. DNA was visualized on agarose gel stained with ethidium bromide, and DNA concentration was measured using the Qubit 2.0 Fluorometer with the dsDNA BR Assay Kit (Life Technologies, Carlsbad, CA, USA) according to the manufacturer’s instructions.

### DNA sequencing and phylogenetic analyses

The internal transcribed spacer (ITS) gene, recognized as the universal DNA barcode for fungi [17], was systematically amplified and sequenced for all isolates. NCBI nucleotide blast of the ITS sequences was performed to detect misidentification and potential contamination of isolates. A list of the different gene regions sequenced along with the primers used is presented in Table 2. PCR reactions were performed in a final volume of 25 μl and contained 1X PCR buffer, 1.5 mM MgCl_2_, 200 μM of each dNTP (Invitrogen), 0.4 μM of each primer (Integrated DNA Technologies Inc., Coralville, IA, USA), 1 U of Platinum *Taq* DNA polymerase (Invitrogen), and 1 μl of template DNA. Sequencing of both DNA strands was performed by the Centre de recherche du Centre Hospitalier Universitaire de Québec (CHUQ) sequencing platform on an ABI 3730xl (Applied Biosystems, Foster City, CA, USA) using the specific forward and reverse primers.

**Table 2:** Primers used for DNA sequencing and genus general assays.

					NCBI Accession Number					
Target gene	Primer name	Amplicon length (bp)	Sequence (5’→ 3’)	Reference	*Ceratocystis*	*Fusarium*	*Geosmithia*	*Gremminiella*	*Rosellinia*	*Sclerotinia*
**DNA sequencing**										
ITS	ITS1F	~ 600	CTTGGTCATTTAGAGGAAGTAA	[70]	KC305097- KC305166	KC464615-KC464634	KF808295-KF808322	KC352952-KC352997	KF719196-KF719202	KF859918-KF859936
	ITS4		TCCTCCGCTTATTGATATGC	[71]						
β-tubulin	T10	~1300	ACGATAGGTTCACCTCCAGAC	[72]	KC335975-KC336019;					
	BT12		GTTGTCAATGCAGAAGGTCTC	[72]	KC589388-KC589393					
EF1	EF1F	~ 900	TGCGGTGGTATCGACAAGCGT	[73]	KC405262-KC405285;					
	EF2R		AGCATGTTGTCGCCGTTGAAG	[73]	KC583303-KC583321					
Tsr1	Tsr1_1453for	~ 900	CCIGAYGARATYGARCTICAYCC	[74]	KC405286-KC405319;					
	Tsr1_2308rev		CTTRAARTAICCRTGIGTICC	[74]	KC590615-KC590632					
IGS	RU46.67	~ 900	GTGTCGGCGTGCTTGTATT	[75]		KC147546-KC147564				
	CNS12		GCACGCCAGGACTGCCTCGT	[75]						
TEF	Ef1	~ 675	ATGGGTAAGGARGACAAGAC	[76]		KC514053-KC514067				
	Ef2		GGARGTACCAGTSATCATGTT	[76]						
β-tubulin	T1	~ 850	AACATGCGTGAGATTGTAAGT	[77]			KF853893-KF853956			
	Bt2b		ACCCTCAGTGTAGTGACCCTTGGC	[78]						
RPB2	RPB2F5	~ 550	CTATACTATCCCCAGAAGCCTCTTGCTACC	This study				KC533095-KC533140		
	RPB2R2		CAATNGTWCCCTTYTGHCCGTGACG	This study						
LSU	LR0R	~ 875	ACCCGCTGAACTTAAGC	[79]					KF719203-KF719215	
	LR5		TCCTGAGGGAAACTTCG	[79]						
Calmodulin	CAL_228F	~ 500	GAGTTCAAGGAGGCCTTCTCCC	[80]						KF871364-KF871386
	CAL_737R		CATCTTTCTGGCCATCATGG	[80]						
G3PDH	G3PDH-Fbis	~ 850	GCTGTCAACGACCCTTTCAT	[81]						KF878354-KF878375
	G3PDH-Rbis		ACCAGGAAACCAACTTGACG	[81]						
HSP60	HSP60for-deg	~ 975	CAACAATTGAGATTYGCCCAYAAG	[81]						KF871387-KF871408
	HSP60rev-deg		GATRGATCCAGTGGTACCGAGCAT	[81]						
**Genus general assay**										
EF1	Cerato_GEN_F510	166	CGTGCTCGCCGGAAATAG	This study						
	Cerato_GEN_R612		TGCCGCCTTTTGGTGC	This study						
IGS	Fus_GEN_F68	119	GCCACCAAACCACAAAACC	This study						
	Fus_GEN_R186		CCCACAGACCTCGCAC	This study						
β-tubulin	Geos_GEN_F479	168	GTAGACGCTCATGCGCTC	This study						
	Geos_GEN_R646		GTAACCAGATCGGTGCTGC	This study						
RPB2	Gremm_GEN_F304	128	CCAATCTGTGGAATCTTCGTGG	This study						
	Gremm_GEN_R431		CGGGATGCTTCAACTCCTC	This study						
LSU	Rosel_GEN_F771	190	CTACTCGACTCGTCGAAGGAG	This study						
	Rosel_GEN_R960		GCGAGTGAAGCGGCAACAG	This study						
Hsp60	Sclero_GEN_F193	178	CTCCCCAAAGATCACCAAAGGTT	This study						
	Sclero_GEN_R371		GGCAACATCTTGAATAAGTCTAGCACC	This study						
β-tubulin	Phyto_GEN_F736	80	GGCTCGCAGCAGTACC	This study						
	Phyto_GEN_R815		GCGGCGCACATCATGTTCT	This study						

Alignments were used to guide the development of assays and were performed with the ClustalW algorithm implemented in BioEdit v7.1.3.0 [18]. Evolutionary relationships between targets and their sister species were inferred from DNA sequences of ITS. Phylogenetic trees were reconstructed by using the maximum likelihood method with the Tamura-Nei model implemented in MEGA5 [19]. Statistical support of nodes was assessed by performing 500 bootstrap replicates.

For the *Phytophthora ramorum* and *Phytophthora kernoviae* targets, we did not perform the gene region sequencing and phylogenetic analyses described above. Instead, the detection assays for these two species were designed in genes unique to these target species that were identified by using a comparative genomics approach developed in the TAIGA project (http://taigaforesthealth.com/Home.aspx) (S1 File).

### SYBRGreen-based real-time PCR quantification for standardization of isolates’ DNA concentration

DNA concentration of all isolates was standardized following a qPCR quantification using genus general primers. To do so, we quantified the number of target gene copies that were initially present (before any PCR amplification) in the sample, which directly relates to the abundance of the pathogen prior to DNA extraction. This quantification allowed us to work with samples having a standardized DNA concentration for specificity validation. It assured us we had DNA in high enough concentration in all samples to confirm assay discrimination against closely related species.

Genus general primers were designed using Oligo Explorer v1.2 and Oligo Analyzer v1.2 (Gene Link, NY, USA) in a conserved gene region for all closely related species. The following criteria were also used to guide primer design: 1) length between 18 and 25 bp; 2) melting temperature (T_m_) close to 60°C (using the nearest neighbor algorithm); 3) absence of polymorphism within targeted species; and 4) minimal secondary structure (especially dimer formation at the 3’ end). Primer pairs were designed such that PCR products were shorter than 200 bp (Table 2). Real-time PCR was performed with an Applied Biosystems 7500 Fast Real-Time PCR System (Life Technologies, Carlsbad, CA, USA). All reactions were performed in a final volume of 10 μl and contained 1X QuantiTect SYBR Green PCR Master Mix (Qiagen, Valencia, CA, USA), 0.5 μM of each of the genus general primers (Table 2), and 1 μl of template DNA. Real-time PCR thermocycling conditions were set at 95°C for 15 min, followed by 50 cycles at 95°C for 15 s, 58°C (primer Tm-2°C) for 30 s, and 65°C for 90 s. Fluorescence was read at the end of the extension step.

Gene copy number quantification was then performed using a Java program based on linear regression of efficiency [20] and sample DNA concentration was adjusted to 5,000 gene copies per μl, whenever possible.

### Target-specific TaqMan-based real-time PCR assays

All the molecular detection assays targeting prioritized tree pathogens are based on the TaqMan technology. The following strategies were used to design all of our detection assays. Based on the sequences recovered, we targeted the gene that allowed for the best discrimination at the species level, i.e. the gene that maximized the number of single nucleotide polymorphisms (SNPs) between species while keeping a low level of intraspecific variability. Primer and probe designs were performed using Oligo Explorer v1.2 and Oligo Analyzer v1.2. Each set of primer pair and probe was designed so that there was minimal secondary structure (especially dimer formation at the 3’ end) and amplicon length did not exceed 350 base pairs (Table 3). Primers and probes were also designed to ascertain that interspecific SNPs were preferentially localized at the 3’ end of the primers for maximum discrimination effect of the primer-template annealing [21]. The real-time PCR master mix used, QuantiTect Multiplex PCR NoROX Master Mix (Qiagen), possesses features that allow for the use of short oligonucleotides when necessary. By allowing the design of shorter primers and probes, these elements increase the SNP specificity of the primer and probe. All probes were labelled with fluorescein (6-FAM) at the 5’ end and with the quencher Iowa Black FQ (ZEN-IBFQ). All primers and TaqMan probes were manufactured by Integrated DNA Technologies Inc. All assays were designed to work under the same thermocycling conditions, offering the opportunity to array them into 96- or 384-well plates machine formats, based on the user’s needs.

**Table 3:** Primers used for the 10 tree pathogen species-specific TaqMan assays.

Name	Target gene	Primer/Probe	Sequence (5’→ 3’)	Amplicon length (bp)
***Ceratocystis laricicola***				
Claricicola_F451	β-tubulin	Forward	GCCCGCATCATGTTT	88
Claricicola_R538		Reverse	GACGCTTGAGCGG	
Claricicola_T505RC		Probe	6-Fam/TGTGCCTGC/ZEN/TCTGATTCAT/3IABkFQ	
***Ceratocystis polonica***				
Cpolonica_F527	β-tubulin	Forward	CGTCCACGCCACAAT	235
Cpolonica_R761		Reverse	CCTGAACACCAATTATGTTATATC	
Cpolonica_T575		Probe	6-Fam/TGTATGATG/ZEN/AGACTAGACGATGC/3IABkFQ	
***Ceratocystis fagacearum***				
Cfagacearum_F315	EF1	Forward	GTCTGTAGAAGGGGG	92
Cfagacearum_R406		Reverse	CTCCATTCTTTACTACAACC	
Cfagacearum_T357		Probe	6-Fam/AGAAGTAAC/ZEN/TGGACAACCGTCT/3IABkFQ	
***Fusarium circinatum***				
Fcircinatum_F656	IGS	Forward	CTATACAGCTTACATAATCATAC	119
Fcircinatum_R775		Reverse	AGGGTAGGCTTGGAT	
Fcircinatum_T717		Probe	6-Fam/TGTCCCTTC/ZEN/TCGAGCCA/3IABkFQ	
***Geosmithia morbida***				
Gmorbida_F677	β-tubulin	Forward	AGTCAGTGTTCTGACC	202
Gmorbida_R878		Reverse	GAAGAAGAATAGGACGG	
Gmorbida_T738		Probe	6-Fam/AATAGGCTG/ZEN/GACAGGAAGA/3IABkFQ	
***Gremmeniella abietina* (EU race)**				
Gabietina_F2b	RPB2	Forward	GGCGCGGTCTTC	216
Gabietina_R4		Reverse	GTATCGATCGTGGTCTA	
Gabietina_T3		Probe	6-Fam/AATGATGTC/ZEN/CTCTCCAGATAC/3IABkFQ	
***Rosellinia necatrix***				
Rnecatrix_F517	ITS	Forward	GGTAGGGCACTTC	102
Rnecatrix_R618		Reverse	GGGATCATTAAAGAGTTCTA	
Rnecatrix_T551		Probe	6-Fam/AGGCAACGCGTGGTAT/3IABkFQ	
***Sclerotinia pseudotuberosa***				
Spseudotuberosa_F218	Hsp60	Forward	TTGTAGAACTCCTAGTCGTA	129
Spseudotuberosa_R347		Reverse	ACCGAGATTCTCGAATTTGTCTTTA	
Spseudotuberosa_T269		Probe	6-Fam/ATCTCTAAT/ZEN/TGTTGTCGAACAGATGGT/3IABkFQ	
***Phytophthora ramorum***				
Pram-C62-F	Cluster62 ^a^	Forward	AACATGCTCGTGCTCAAGTG	116
Pram-C62-R		Reverse	CGGTGTTCTGGCGTTCTAGT	
Pram-C62-P		Probe	6-Fam/CAAGGGGAC/ZEN/CGGAACCGTAT/3IABKFQ	
***Phytophthora kernoviae***	^a^			
Pkernoviae_F97	Cluster97	Forward	GGACTGTGCAGCGCCTAT	112
Pkernoviae_R97		Reverse	TCATCACCCCATTTCTTGC	
Pkernoviae_T97		Probe	6-Fam/TGCCTCACC/ZEN/ACCAGATGG/3IABKFQ	
**Plant DNA extraction control**				
PLCOIF57-74	Cytochrome oxidase	Forward	TAAACATATGATGAGCCC	184
PLCOIR223-240		Reverse	AGCATCTCTTTTGGTTCT	
PLCOIT98-120		Probe	6-Fam/ATACTGATCATGGCATAAACCAT/3IABKFQ	
**Insect DNA extraction control**				
InsectF1418	28S rRNA gene	Forward	CCAAGGAGTCTAGCAT	264
InsectR1681		Reverse	GGTCCCAGCGTGT	
InsectT1595		Probe	6-Fam/TTCCCGGGGCGTCTC/3IABKFQ	

^a^ *P*. *ramorum* Cluster62 and *P*. *kernoviae* Cluster97 are both hypothetical proteins without any known function so far.

The validation principles and parameters followed the terminology and concepts described in Charlton (2000) [22] and Ederveen (2010) [23].

### Validating the specificity of the tree pathogen TaqMan assays

**Fig 1:**
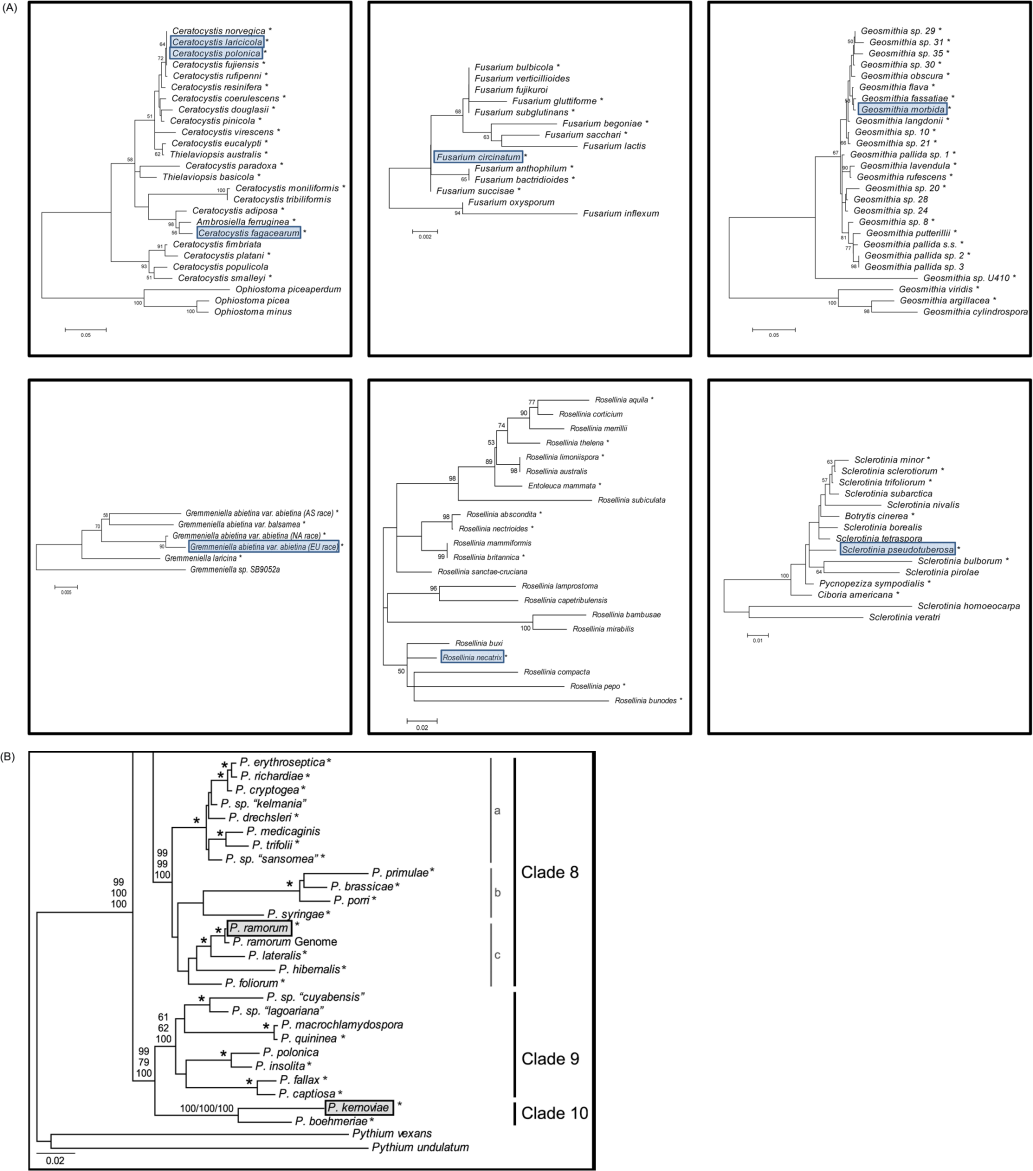
Phylogenetic trees of each genus, including target and closely related species. For each tree, the target species is (are) shaded. Species followed by an asterisk (*) were used to perform specificity validation. (A) Maximum likelihood phylogenetic trees using internal transcribed spacer (ITS) sequences. (B) Maximum likelihood phylogenetic tree of *Phytophthora* clades 8–10 using seven nuclear loci (from Blair *et al*. [15]).

Specificity validation of all the assays was performed using the panels of isolates presented in Table 1 and Fig 1. For target species belonging to same genera (*Ceratocystis* and *Phytophthora*), we used the whole genera panel to evaluate each of the assay’s specificity. Real-time PCR amplification was conducted using 1X QuantiTect Multiplex PCR NoROX Master Mix, with 0.6 μM of each primer, 0.1 μM of TaqMan probe, and 5,000 gene copies of template DNA, whenever possible, in a final reaction volume of 10 μl. Two technical replicates were performed for all reactions using an Applied Biosystems 7500 Fast Real-Time PCR System. Real-time PCR thermocycling conditions were set at 95°C for 15 min, followed by 50 cycles at 95°C for 15 s and 60°C for 90 s. Fluorescence was read at each cycle, at the end of the extension step.

### Validating the sensitivity of the tree pathogen TaqMan assays

Sensitivity of the TaqMan assays was evaluated in terms of both efficiency and limit of detection (LOD). For each target assay, experiments were conducted to 1) determine if Ct values were proportional to the amount of target template DNA (efficiency) and 2) evaluate the LOD, which is the smallest amount of target DNA that can be detected for each of the assays. One isolate for each of the target species was selected, and TaqMan assay sensitivity was assessed on parallel sets of serial dilutions from the DNA stock.

To assess efficiency of the amplification reaction, TaqMan assays were run with serial dilutions of template DNA from the target species, ranging from 1 to 15,000 copies of the target gene region, as quantified using the species-specific primers. Standard curves were obtained by plotting the values of Ct against the log value of the target gene region copy number. Amplification reaction efficiency was calculated using the following formula:

$$E=\left(10^{\left(–1/slope\right)}–1\right)\times 100$$

where *E* represents the amplification reaction efficiency and *slope* is the slope value of the line derived from the standard curve plot. Estimation of the LOD was done by performing 20 replicates of the TaqMan real-time PCR reactions for each of the following DNA concentrations: 1, 3, 5, and 10 copies per μL. The lowest DNA concentration with a level of 95% successful amplification was identified as the LOD.

### Validating the precision of the tree pathogen TaqMan assays

Precision (or repeatability) of the assays refers to the robustness of the assay with the same samples repeatedly analyzed in the same manner [24]. Ct values from different real-time PCR runs on different isolates of target species, assessed with a standardized concentration of 5,000 gene copies, were compiled and used to determine the precision of the assays. For each assay, mean Ct value, standard deviation and coefficient of variation were calculated.

### Validating the tree pathogen TaqMan assays on environmental samples

The complete list of all environmental samples, including the source, is presented in Table 4. Because of the phytosanitary risks of infected material, environmental samples were supplied by collaborators as purified DNA samples. Since the objective was to test the assays’ performance in a variety of different conditions, collaborators were free to use the routine DNA extraction protocols implemented in their respective laboratories instead of a unique standardized DNA extraction protocol. The efficiency of the DNA extraction was assessed for each sample by performing a control real-time TaqMan PCR reaction that targeted either the plant cytochrome oxidase gene (for primers and probes sequences, see Table 3). All reactions were performed in a final volume of 10 μL and contained 1X QuantiTect Multiplex PCR NoROX Master Mix, with 0.6 μM of each primer, 0.1 μM of TaqMan probe, and 1 μL of template DNA. Real-time PCR thermocycling conditions were set at 95°C for 15 min, followed by 50 cycles at 95°C for 15 s and 60°C for 90 s. Fluorescence was read at the end of each cycle.

**Table 4:** Description of environmental samples.

Isolate	Type of material^a^	Host	Location	Year of collection	Collector/Provider
***Ceratocystis laricicola*** ^b^					
CEM5	Juvenile or adult *Ips cembrae* collected from galleries	European larch (*Larix decidua*)	Austria	2010	T. Kirisits
CEM8	Juvenile or adult *Ips cembrae* collected from galleries	European larch (*Larix decidua*)	Austria	2010	T. Kirisits
CEM10	Juvenile or adult *Ips cembrae* collected from galleries	European larch (*Larix decidua*)	Austria	2010	T. Kirisits
CEM11	Juvenile or adult *Ips cembrae* collected from galleries	European larch (*Larix decidua*)	Austria	2010	T. Kirisits
CEM13	Juvenile or adult *Ips cembrae* collected from galleries	European larch (*Larix decidua*)	Austria	2010	T. Kirisits
CEM19	Juvenile or adult *Ips cembrae* collected from galleries	European larch (*Larix decidua*)	Austria	2010	T. Kirisits
CEM25	Juvenile or adult *Ips cembrae* collected from galleries	European larch (*Larix decidua*)	Austria	2010	T. Kirisits
***Ceratocystis polonica*** ^b^					
TYP1	Adult *Ips typographus* collected from galleries	Norway spruce (*Picea abies*)	Austria	2010	T. Kirisits
TYP2	Adult *Ips typographus* collected from galleries	Norway spruce (*Picea abies*)	Austria	2010	T. Kirisits
TYP3	Adult *Ips typographus* collected from galleries	Norway spruce (*Picea abies*)	Austria	2010	T. Kirisits
TYP11	Adult *Ips typographus* collected from galleries	Norway spruce (*Picea abies*)	Austria	2010	T. Kirisits
TYP16	Adult *Ips typographus* collected from galleries	Norway spruce (*Picea abies*)	Austria	2010	T. Kirisits
TYP17	Adult *Ips typographus* collected from galleries	Norway spruce (*Picea abies*)	Austria	2010	T. Kirisits
TYP19	Adult *Ips typographus* collected from galleries	Norway spruce (*Picea abies*)	Austria	2010	T. Kirisits
***Ceratocystis fagacearum***					
SAP-1	Sapwood of infected host	Red oak (*Quercus rubra*)	MN, USA	2014	J. Juzwik
SAP-2	Sapwood of infected host	Red oak (*Quercus rubra*)	MN, USA	2014	J. Juzwik
SAP-3	Sapwood of infected host	Red oak (*Quercus rubra*)	MN, USA	2014	J. Juzwik
SAP-4	Sapwood of infected host	Red oak (*Quercus rubra*)	MN, USA	2014	J. Juzwik
SAP-5	Sapwood of infected host	Red oak (*Quercus rubra*)	MN, USA	2014	J. Juzwik
SAP-6	Sapwood of infected host	Red oak (*Quercus rubra*)	MN, USA	2014	J. Juzwik
CS1	*Carpophilus sayi* collected from oak wilt mats	Red oak (*Quercus rubra*)	MN, USA	2014	J. Juzwik
CS2	*Carpophilus sayi* collected from oak wilt mats	Red oak (*Quercus rubra*)	MN, USA	2014	J. Juzwik
CS3	*Carpophilus sayi* collected from oak wilt mats	Red oak (*Quercus rubra*)	MN, USA	2014	J. Juzwik
CS4	*Carpophilus sayi* collected from oak wilt mats	Red oak (*Quercus rubra*)	MN, USA	2014	J. Juzwik
CS5	*Carpophilus sayi* collected from oak wilt mats	Red oak (*Quercus rubra*)	MN, USA	2014	J. Juzwik
CS6	*Carpophilus sayi* collected from oak wilt mats	Red oak (*Quercus rubra*)	MN, USA	2014	J. Juzwik
CS7	*Carpophilus sayi* collected from oak wilt mats	Red oak (*Quercus rubra*)	MN, USA	2014	J. Juzwik
CS8	*Carpophilus sayi* collected from oak wilt mats	Red oak (*Quercus rubra*)	MN, USA	2014	J. Juzwik
CS9	*Carpophilus sayi* collected from oak wilt mats	Red oak (*Quercus rubra*)	MN, USA	2014	J. Juzwik
EC1	*Epuraea corticina* collected from oak wilt mats	Red oak (*Quercus rubra*)	MN, USA	2014	J. Juzwik
EC2	*Epuraea corticina* collected from oak wilt mats	Red oak (*Quercus rubra*)	MN, USA	2014	J. Juzwik
EC3	*Epuraea corticina* collected from oak wilt mats	Red oak (*Quercus rubra*)	MN, USA	2014	J. Juzwik
GS1	*Glischrochilus sanguinolentus* collected from oak wilt mats	Red oak (*Quercus rubra*)	MN, USA	2014	J. Juzwik
GS2	*Glischrochilus sanguinolentus* collected from oak wilt mats	Red oak (*Quercus rubra*)	MN, USA	2014	J. Juzwik
GS3	*Glischrochilus sanguinolentus* collected from oak wilt mats	Red oak (*Quercus rubra*)	MN, USA	2014	J. Juzwik
GS4	*Glischrochilus sanguinolentus* collected from oak wilt mats	Red oak (*Quercus rubra*)	MN, USA	2014	J. Juzwik
GS5	*Glischrochilus sanguinolentus* collected from oak wilt mats	Red oak (*Quercus rubra*)	MN, USA	2014	J. Juzwik
***Fusarium circinatum***					
SB1a	Woody tissue of asymptomatic host	Monterey pine (*Pinus radiata*)	CA, USA	N/A	R. Ioos
SB3a	Woody tissue of asymptomatic host	Monterey pine (*Pinus radiata*)	CA, USA	N/A	R. Ioos
SB4a	Woody tissue of asymptomatic host	Monterey pine (*Pinus radiata*)	CA, USA	N/A	R. Ioos
71-1A	Woody tissue	Loblolly pine (*Pinus taeda*)	USA	N/A	R. Ioos
77-1A	Woody tissue	Ponderosa pine (*Pinus ponderosa*)	USA	N/A	R. Ioos
124a	Woody tissue	Loblolly pine (*Pinus taeda*)	USA	N/A	R. Ioos
819A	Woody tissue	Loblolly pine (*Pinus taeda*)	USA	N/A	R. Ioos
860B	Woody tissue	Maritime pine (*Pinus pinaster*)	Spain	N/A	R. Ioos
MP1Ab	Woody tissue of symptomatic host	Monterey pine (*Pinus radiata*)	CA, USA	N/A	R. Ioos
MP1Ba	Woody tissue of asymptomatic host	Monterey pine (*Pinus radiata*)	CA, USA	N/A	R. Ioos
MP2A	Woody tissue of symptomatic and asymptomatic host	Monterey pine (*Pinus radiata*)	CA, USA	N/A	R. Ioos
MP3a	Woody tissue of symptomatic and asymptomatic host	Monterey pine (*Pinus radiata*)	CA, USA	N/A	R. Ioos
MP4Ba	Woody tissue of symptomatic host	Monterey pine (*Pinus radiata*)	CA, USA	N/A	R. Ioos
MP5Aa	Woody tissue of symptomatic host	Monterey pine (*Pinus radiata*)	CA, USA	N/A	R. Ioos
MP5Ba	Woody tissue of asymptomatic host	Monterey pine (*Pinus radiata*)	CA, USA	N/A	R. Ioos
MP6a	Woody tissue of symptomatic and asymptomatic host	Monterey pine (*Pinus radiata*)	CA, USA	N/A	R. Ioos
MP7a	Woody tissue of symptomatic and asymptomatic host	Monterey pine (*Pinus radiata*)	CA, USA	N/A	R. Ioos
S10-14	*Ips sexdentatus* artificially inoculated with 10 spores of *F*. *circinatum*	-	-	-	R. Ioos
S50-13	*Ips sexdentatus* artificially inoculated with 50 spores of *F*. *circinatum*	-	-	-	R. Ioos
S100-15	*Ips sexdentatus* artificially inoculated with 100 spores of *F*. *circinatum*	-	-	-	R. Ioos
***Geosmithia morbida***					
JN2 Poz	Artificially-inoculated host (greenhouse)	Eastern black walnut (*Juglans nigra*)	-	-	M. Kolařík
JN3 Neg	Non-inoculated host (greenhouse)	Eastern black walnut (*Juglans nigra*)	-	-	M. Kolařík
WTB-G3-1	*Pityophthorus juglandis* from TN, USA	Eastern black walnut (*Juglans nigra*)	TN, USA	2013	J. Juzwik
WTB-G3-2	*Pityophthorus juglandis* from TN, USA	Eastern black walnut (*Juglans nigra*)	TN, USA	2013	J. Juzwik
WTB-G3-3	*Pityophthorus juglandis* from TN, USA	Eastern black walnut (*Juglans nigra*)	TN, USA	2013	J. Juzwik
WTB-G3-4	*Pityophthorus juglandis* from TN, USA	Eastern black walnut (*Juglans nigra*)	TN, USA	2013	J. Juzwik
WTB-G3-5	*Pityophthorus juglandis* from TN, USA	Eastern black walnut (*Juglans nigra*)	TN, USA	2013	J. Juzwik
WTB-G3-6	*Pityophthorus juglandis* from TN, USA	Eastern black walnut (*Juglans nigra*)	TN, USA	2013	J. Juzwik
WTB-G3-7	*Pityophthorus juglandis* collected on host	Eastern black walnut (*Juglans nigra*)	TN, USA	2013	J. Juzwik
WTB-G3-8	*Pityophthorus juglandis* collected on host	Eastern black walnut (*Juglans nigra*)	TN, USA	2013	J. Juzwik
WTB-G3-9	*Pityophthorus juglandis* collected on host	Eastern black walnut (*Juglans nigra*)	TN, USA	2013	J. Juzwik
WTB-G3-10	*Pityophthorus juglandis* collected on host	Eastern black walnut (*Juglans nigra*)	TN, USA	2013	J. Juzwik
WTB-G10-1	*Pityophthorus juglandis* collected on host	Eastern black walnut (*Juglans nigra*)	TN, USA	2013	J. Juzwik
WTB-G10-2	*Pityophthorus juglandis* collected on host	Eastern black walnut (*Juglans nigra*)	TN, USA	2013	J. Juzwik
WTB-G10-3	*Pityophthorus juglandis* collected on host	Eastern black walnut (*Juglans nigra*)	TN, USA	2013	J. Juzwik
WTB-G10-4	*Pityophthorus juglandis* collected on host	Eastern black walnut (*Juglans nigra*)	TN, USA	2013	J. Juzwik
WTB-G10-5	*Pityophthorus juglandis* collected on host	Eastern black walnut (*Juglans nigra*)	TN, USA	2013	J. Juzwik
WTB-G10-6	*Pityophthorus juglandis* collected on host	Eastern black walnut (*Juglans nigra*)	TN, USA	2013	J. Juzwik
WTB-G10-7	*Pityophthorus juglandis* collected on host	Eastern black walnut (*Juglans nigra*)	TN, USA	2013	J. Juzwik
WTB-G10-8	*Pityophthorus juglandis* collected on host	Eastern black walnut (*Juglans nigra*)	TN, USA	2013	J. Juzwik
WTB-G10-9	*Pityophthorus juglandis* collected on host	Eastern black walnut (*Juglans nigra*)	TN, USA	2013	J. Juzwik
WTB-G10-10	*Pityophthorus juglandis* collected on host	Eastern black walnut (*Juglans nigra*)	TN, USA	2013	J. Juzwik
***Gremmeniella abietina* (EU race)**					
64667	Needles	Jack pine (*Pinus banksianae*)	QC, Canada	2013	MRNQ
64668	Needles	Jack pine (*Pinus banksianae*)	QC, Canada	2013	MRNQ
64672	Needles	Jack pine (*Pinus banksianae*)	QC, Canada	2013	MRNQ
64673	Needles	Jack pine (*Pinus banksianae*)	QC, Canada	2013	MRNQ
65097	Needles	Red pine (*Pinus resinosa*)	QC, Canada	2013	MRNQ
65171	Needles	Red pine (*Pinus resinosa*)	QC, Canada	2013	MRNQ
65181	Needles	Jack pine (*Pinus banksianae*)	QC, Canada	2013	MRNQ
65539	Needles	Jack pine (*Pinus banksianae*)	QC, Canada	2013	MRNQ
67161	Needles	Red pine (*Pinus resinosa*)	QC, Canada	2013	MRNQ
***Rosellinia necatrix***					M. Shishido
A	Roots	Japanese pear (*Pyrus pyrifolia* var. *culta*)	Japan	N/A	M. Shishido
B	Roots	Japanese pear (*Pyrus pyrifolia* var. *culta*)	Japan	N/A	M. Shishido
D	Roots	Japanese pear (*Pyrus pyrifolia* var. *culta*)	Japan	N/A	M. Shishido
E	Roots	Japanese pear (*Pyrus pyrifolia* var. *culta*)	Japan	N/A	M. Shishido
H	Roots	Japanese pear (*Pyrus pyrifolia* var. *culta*)	Japan	N/A	M. Shishido
I	Roots	Japanese pear (*Pyrus pyrifolia* var. *culta*)	Japan	N/A	M. Shishido
***Sclerotinia pseudotuberosa***					
1C	Nuts	Sweet chestnut (*Castanea sativa*)	Italy	N/A	G. Maresi
2C	Nuts	Sweet chestnut (*Castanea sativa*)	Italy	N/A	G. Maresi
***Phytophthora ramorum***					
16883	N/A	N/A	UK	N/A	J. Tomlinson
16885	N/A	N/A	UK	2007	J. Tomlinson
17085	Leaves	Rhododendron (*Rhododendron* sp.)	UK	2010	J. Tomlinson
17385	Leaves	Chinese magnolia (*Magnolia* x *soulangeana*)	UK	2008	J. Tomlinson
17358	Leaves	*Griselinia* sp.	UK	2008	J. Tomlinson
07-Qr3-2i	Leaf of artificially-inoculated host (greenhouse)	Red oak (*Quercus rubra*)	-	-	D. Rioux
07-Ab3-1i	Leaf of artificially-inoculated host (greenhouse)	Balsam fir (*Abies balsamea*)	-	-	D. Rioux
07-As2-4i	Leaf of artificially-inoculated host (greenhouse)	Sugar maple (*Acer saccharum*)	-	-	D. Rioux
07-Ll1-3i	Leaf of artificially-inoculated host (greenhouse)	Tamarack (*Larix laricina*)	-	-	D. Rioux
07-Fa3-1i	Leaf of artificially-inoculated host (greenhouse)	White ash (*Fraxinus Americana*)	-	-	D. Rioux
07-Ba1-2i	Leaf of artificially-inoculated host (greenhouse)	Yellow birch (*Betula alleghaniensis*)	-	-	D. Rioux
07-Rho1-4i	Leaf of artificially-inoculated host (greenhouse)	Rhododendron (*Rhododendron catawbiense* cv. *Nova zembla*)	-	-	D. Rioux
07-Rho1-2c	Leaf of non-inoculated host (greenhouse)	Rhododendron (*Rhododendron catawbiense* cv. *Nova zembla*)	-	-	D. Rioux
02045	N/A	N/A	UK	2011	J. Tomlinson
19347	Leaf litter/soil	N/A	UK	2011	J. Tomlinson
20181	Water bait	N/A	UK	2011	J. Tomlinson
20644	Leaves	Rhododendron (*Rhododendron* sp.)	UK	2011	J. Tomlinson
20816	Water bait	N/A	UK	2011	J. Tomlinson
***Phytophthora kernoviae***					
16833	N/A	N/A	UK	N/A	J. Tomlinson
16876	Leaves	Rhododendron (*Rhododendron* sp.)	UK	2007	J. Tomlinson
17072	N/A	N/A	UK	N/A	J. Tomlinson
02045	N/A	N/A	UK	2011	J. Tomlinson
19347	Leaf litter/soil	N/A	UK	2011	J. Tomlinson
20181	Water bait	N/A	UK	2011	J. Tomlinson
20644	N/A	Rhododendron (*Rhododendron* sp.)	UK	2011	J. Tomlinson
20816	Water bait	N/A	UK	2011	J. Tomlinson

^a^ Type of material from which DNA was extracted.

^b^ TYP samples were used as negative controls for *C*. *laricicola* specific assays, whereas CEM samples were used as negative controls for *C*. *polonica* specific assays.

TaqMan assays were performed with three technical replicates for each related environmental sample. Reactions were performed as described earlier, using 1 μL of environmental DNA. Positive (using target species’ DNA from pure culture) and negative (no template DNA) controls were included in all qPCR runs. Target gene region copy numbers were calculated by translating Ct values, using standard curve equations. Positive results were all confirmed by Sanger sequencing of the real-time TaqMan PCR product.

## Results and Discussion

### Assay design and development

Development of the detection assays was based on two strategies targeting 1) unique SNPs or 2) unique genes. The SNP-based approach uses alignment of genes present in all species, but exploits the presence of SNPs between the target species and the close relatives. It was used for all assays reported in this paper except for *P*. *ramorum* and *P*. *kernoviae*. For both of these species, comparative genomics was used to identify genes uniquely found in the target species to design the detection assay. Detail about this TAIGA strategy, the related genomic resources and bioinformatics pipeline are available on the TAIGA project website (http://taigaforesthealth.com/).

A crucial step in the development of the SNP-based detection assays is the identification of appropriate target DNA regions. Readily amplified genes across taxa of a group were sequenced, and genes showing interspecific variability were selected for assay development (Table 2). As a result, different genes were selected for each target species, some of them being single- or low-copy genes (e.g. β-tubulin, EF1, RPB2 and Hsp60), and others being multi-copy genes (e.g. IGS, ITS, Cluster62 and Cluster97).

In order to standardize the DNA concentration of all isolates, a genus general real-time PCR SYBRGreen I assay targeting the selected DNA region was designed and validated for each target group. Using the linear regression of efficiency (LRE) quantification approach [20], DNA concentration of isolates was determined and standardized to 5,000 target gene region copies, which usually translates into a Ct value ranging between 20 and 25.

To ensure the repeatability of the qPCR experiments described by other teams and to ease the interpretation of results, most of the Minimum Information for Quantitative Real-Time PCR Experiments (MIQE), as described by Bustin *et al*. (2009) [24], is presented in this paper.

### Specificity of the tree pathogen TaqMan assays

Probes’ and primers’ specificity was first tested *in silico* using BLAST on the NCBI nucleotide collection (nr/nt) database. A wet lab was then performed to assess candidate sets’ panel specificity on DNA samples from target and sister species isolates. During the first round of specificity validation, whenever an unexpected amplification was observed, two hypotheses were explored. First, it could be due to trace contamination of the DNA sample with target species’ DNA, which sometimes happens during sample manipulation. This was suspected when cycle threshold (Ct) values were much higher (around 35–37) than those of the target species isolates. When contamination was suspected, a SYBRGreen real-time PCR reaction along with a melting curve gradient was performed. When melting curves of positive and suspected false positive samples were identical, the SYBRGreen real-time PCR reaction product of the false positive was sequenced and aligned with reference sequences to confirm the contamination. In such cases, the contaminated DNA sample was discarded and fresh DNA was re-extracted from a pure culture of the isolate. If the first hypothesis was confirmed, we concluded we had a real false positive reaction due to a lack of specificity. In such cases, further screening of primers and probes was conducted.

All our final assays were 100% specific successfully discriminating the target species of tree pathogen from the closely related species. In cases where we were unable to obtain culture or DNA of some closely related species, we still performed *in silico* specificity validation using sequences obtained from the public domain. Despite the current results, we cannot rule out potential cross reactivity of the present assays with evolutionarily related species that have not been described yet. Some of the target species belong to what can be considered as orphan and poorly resolved taxonomic groups (such as *Mycosphaerella*, with newly described species *M*. *musivoides* P.E. Busby & G. Newc and *M*. *wasatchii* P.E. Busby & G. Newc [25]). Instead of being a drawback, molecular cross reactivity with cryptic species can represent an opportunity to isolate and describe novel fungal species with similar or different pathogenic behaviors [26].

### Sensitivity of the tree pathogen TaqMan assay

**Fig 2:**
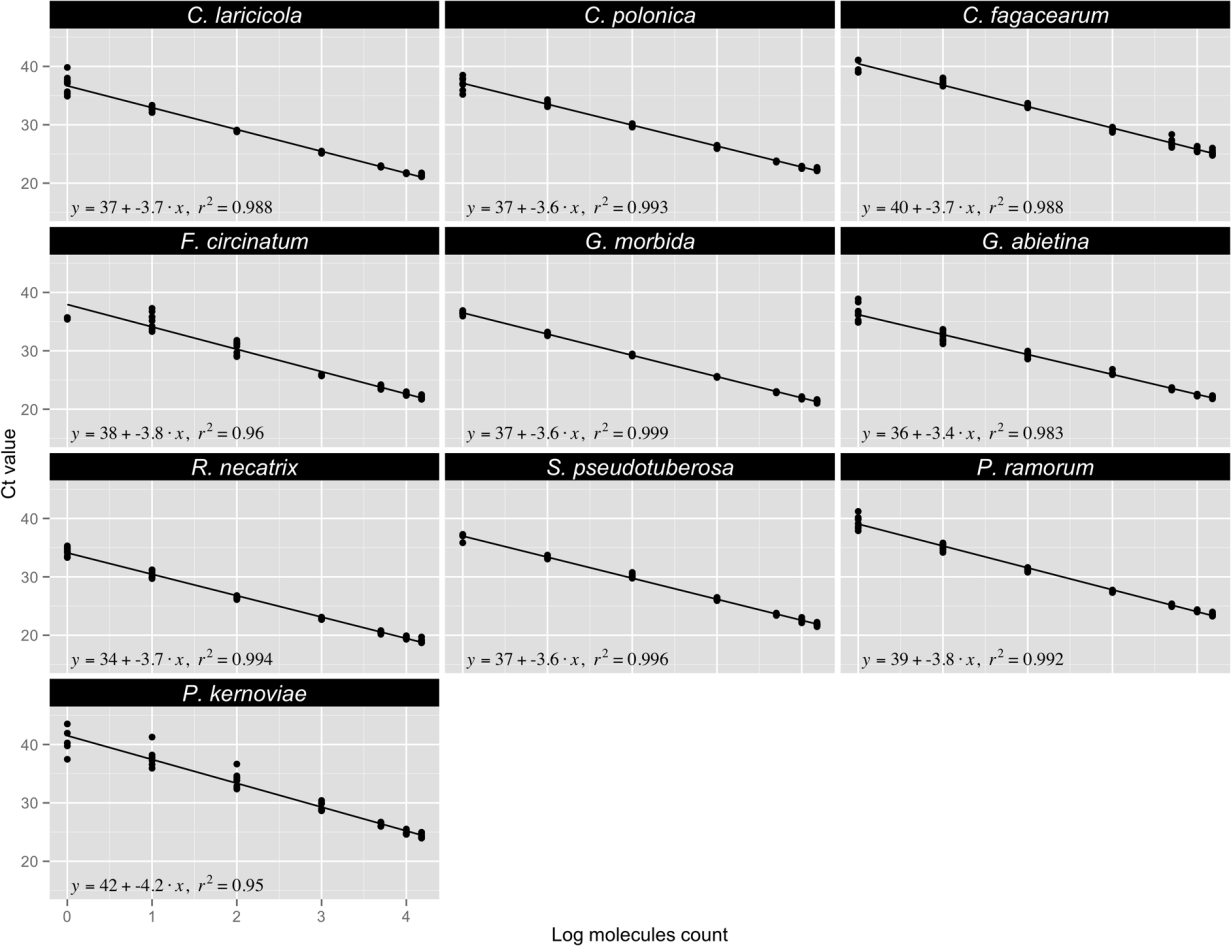
Standard curve for each of the 10 tree pathogen assays. Ct values are plotted against the log value of the target gene region copy number. Curve equations and the squared correlation coefficient are presented.

Overall, the assays we developed have high efficiency and sensitivity, with limits of detection varying between 1 and 10 target gene region copies (Table 5). In the present study, no difference in sensitivity values was observed between assays targeting single- or low-copy genes and those targeting multi-copy genes. For all tested species, Ct values were proportional to the amount of template DNA used for the real-time PCR reaction. The standard curves generated by plotting the log of DNA (copies) against the Ct value determined by qPCR display linearity across the whole range of dilutions assessed, with a correlation coefficient (*r* ^*2*^) ranging from 0.950 for the *P*. *kernoviae* assay to 0.999 for the *G*. *morbida* assay (Fig 2). Moreover, PCR amplification efficiencies ranged between 83 and 97%, which is considered to be an acceptable range [27], except for *P*. *kernoviae*. The low amplification efficiency (73%) of the *P*. *kernoviae* assay could be due to a number of factors, such as the presence of inhibitors in the DNA samples, suboptimal primer and probe design (e.g. presence of non-specific products and primer dimers), and pipetting errors. Experimental investigations ruled out the presence of non-specific products and dimers, sample contamination, inappropriate dilution series and pipetting errors. Another possible explanation for this reduced efficiency is the presence of a secondary structure in the region targeted by this assay. Amplification efficiency can vary across a genome [28, 29]. Genomic regions resistant to amplification by PCR correlate with high GC contents [30, 31] that do not denature efficiently under routine amplification conditions. However, the GC content of that region was around 55%, which is not considered a problem.

**Table 5:** Limit of detection for the 10 tree pathogen TaqMan assays.

Species	Target gene	Limit of detection (LOD)^a^
***Ceratocystis laricicola***	β-tubulin	3
***Ceratocystis polonica***	β-tubulin	3
***Ceratocystis fagacearum***	EF1	10
***Fusarium circinatum***	IGS	10
***Geosmithia morbida***	β-tubulin	3
***Gremmeniella abietina* (EU race)**	RPB2	1
***Rosellinia necatrix***	ITS	1
***Sclerotinia pseudotuberosa***	Hsp60	5
***Phytophthora ramorum***	Cluster62	5
***Phytophthora kernoviae***	Cluster97	3

^a^ Represented as the copy number of the target gene region.

The limit of detection (LOD) was defined as the lowest concentration of target DNA at which 95% of the positive samples were detected [24]. According to Bustin *et al*. (2009), and assuming an even Poisson distribution, the lowest theoretically possible detection limit is three DNA copies per PCR reaction to provide a positive signal in 95% of the PCR reactions performed. Our assays revealed LOD values varying between 1 and 10 target gene region copies (Table 5). Those values are comparable to what has been reported by others working on trace detection for regulatory or public health applications, looking either for the presence of genetically modified DNA [32–35], virus DNA in blood samples [36, 37], or human DNA [38, 39], where LOD values varying between 1 and 25 copies were obtained.

As an additional validation step, we were able to compare results from our *F*. *circinatum* assay with those published for a similar qPCR test developed by Ioos *et al*. (2009) [40]. The published assay was not compliant with our set of real-time PCR conditions and had to be redesigned to suit qPCR standardized parameters. Our assay targets a different segment of the intergenic spacer region than the one used elsewhere [40]. To conduct a fair comparison, a subset of the environmental samples used by Ioos *et al*. (2009) [40] was obtained and tested with our assay. The results we obtained were actually very similar. Although our assay had a slightly delayed detection threshold of approximately 3 Ct values compared with that of Ioos *et al*. (2009), these values were not significantly different.

### Precision of the tree pathogen TaqMan assays

**Fig 3:**
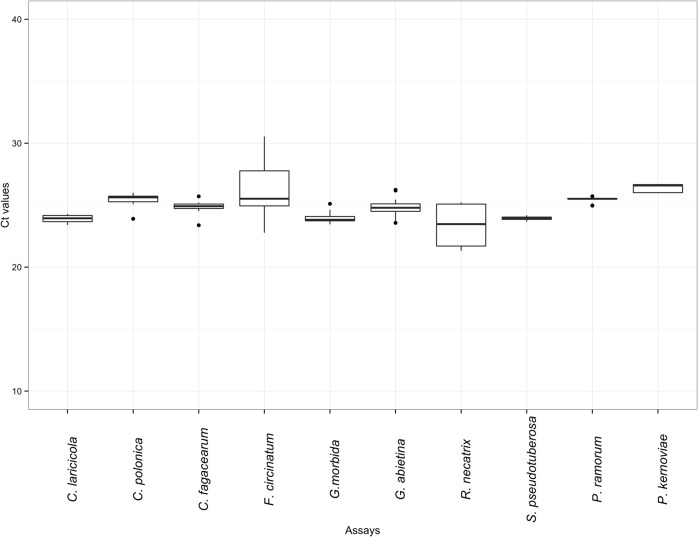
Precision of each of the 10 tree pathogen assays. Box plot representing variation of the Ct value between technical replicates of various isolates from target species (mean Ct value ± coefficient of variation). DNA samples at a concentration of 5,000 copies of the target gene region were used.

The precision of our ten tree pathogen TaqMan assays is shown in Fig 3. All assays have a mean Ct value ranging between 23 and 26 for 5,000 target gene region copies. This value depends on amplicon size and primers and probe properties. Using the mean Ct value and the standard deviation, we also calculated a coefficient of variation for each assay, which varied between 0.7% for the *S*. *pseudotuberosa* assay and 8.2% for the *F*. *circinatum* assay. Those values clearly demonstrate that our assays have a high degree of repeatability, an important advantage when dealing with possible regulatory issues.

### Validation of the tree pathogen TaqMan assays on environmental samples

All tree pathogen assays successfully detected target pathogens from the positive environmental samples provided by collaborators (Tables 4 and 6). Negative environmental DNA samples were available for 6 out of the 10 assays; no false positive results were obtained with any of these.

**Table 6:** Results from species-specific TaqMan real-time PCR assays using environmental samples.

Isolate	Expected result^a^	Genus gene copy number^b^	Specific TaqMan assay Ct value (± SD)	Target gene region copy number^c^
***Ceratocystis laricicola***	+			
CEM5	+	712	29.3 (0.1)	121
CEM8	+	1,701	29.7 (0.1)	97
CEM10	+	542	29.4 (0.2)	117
CEM11	+	1,621	27.9 (0.1)	284
CEM13	+	1,638	28.0 (0.2)	271
CEM19	+	2,227	29.4 (0.1)	111
CEM25	+	964	28.3 (0.1)	220
TYP1	-	910	None	-
TYP2	-	818	None	-
TYP3	-	751	None	-
TYP11	-	555	None	-
TYP16	-	373	None	-
TYP17	-	613	None	-
TYP19	-	176	None	-
***Ceratocystis polonica***				
TYP1	+	910	34.7 (0.4)	4
TYP2	+	818	35.0 (0.5)	4
TYP3	+	751	35.3 (0.3)	3
TYP11	+	555	34.6 (0.1)	5
TYP16	+	373	31.8 (ND)^d^	28
TYP17	+	613	34.6 (0.1)	5
TYP19	+	176	36.1 (ND)^d^	2
CEM5	-	712	None	-
CEM8	-	1,701	None	-
CEM10	-	542	None	-
CEM11	-	1,621	None	-
CEM13	-	1,638	None	-
CEM19	-	2,227	None	-
CEM25	-	964	None	-
***Ceratocystis fagacearum***				
SAP-1	+	148	36.8 (1.9)	7
SAP-2	+	5	37.4 (0.9)	5
SAP-3	+	91	36.5 (0.3)	9
SAP-4	+	54	39.1 (ND)^d^	2
SAP-5	+	2	37.1 (0.6)	6
SAP-6	+	1	38.1 (0.3)	3
CS1	+	4,626	26.5 (0.2)	4,326
CS2	+	3,467	25.8 (0.2)	6,805
CS3	+	2,593	27.5 (0.0)	2,409
CS4	+	3,342	27.4 (0.1)	2,518
CS5	+	4,872	27.3 (0.0)	2,682
CS6	+	2,261	28.1 (0.1)	1,694
CS7	+	3,293	25.6 (0.1)	8,041
CS8	+	2,677	24.7 (0.3)	12,421
CS9	+	1,334	24.5 (0.1)	15,351
EC1	+	7,081	27.0 (0.1)	3,195
EC2	+	8,918	26.9 (0.0)	3,478
EC3	+	13,140	28.3 (0.1)	1,476
GS1	+	6,724	25.8 (0.1)	6,794
GS2	+	2,838	27.5 (0.1)	2,361
GS3	+	3,313	27.2 (0.1)	2,940
GS4	+	6,087	27.2 (0.0)	2,902
GS5	+	41,882	22.7 (0.1)	48,405
***Fusarium circinatum***				
SB1a	+	1	None	-
SB3a	+	6	34.0 (0.8)	11
SB4a	+	300,332	18.1 (0.0)	170,090
71-1A	+	1	None	-
77-1A	+	45	34.5 (0.1)	8
124a	+	1,936	28.0 (0.1)	434
819A	+	8	35.9 (ND)^d^	4
860B	+	262	28.4 (0.1)	332
MP1Ab	+	6,096	24.0 (0.1)	4,944
MP1Ba	+	737	27.0 (0.3)	771
MP2A	+	200	30.1 (0.2)	118
MP3a	+	700	27.0 (0.1)	795
MP4Ba	+	346	27.9 (0.4)	455
MP5Aa	+	49	31.2 (0.0)	63
MP5Ba	+	35	31.6 (0.9)	48
MP6a	+	1,399	26.5 (0.1)	1,087
MP7a	+	551	27.5 (0.0)	588
S10-14	+	1	34.8 (ND)^d^	7
S50-13	+	1	36.0 (ND)^d^	3
S100-15	+	5	32.7 (0.4)	24
***Geosmithia morbida***				
JN2 Poz	+	4	31.7 (0.3)	29
JN3 Neg	-	23	None	-
WTB-G3-1	+	1	34.5 (0.3)	5
WTB-G3-2	+	4	34.5 (0.2)	5
WTB-G3-3	+	1	35.7 (1.0)	2
WTB-G3-4	+	3	34.2 (0.2)	6
WTB-G3-5	+	1	34.9 (0.4)	4
WTB-G3-6	+	13	31.7 (0.1)	30
WTB-G3-7	+	3	34.7 (0.2)	4
WTB-G3-8	+	11	32.5 (0.1)	17
WTB-G3-9	+	2	35.2 (0.7)	3
WTB-G3-10	+	6	33.2 (0.5)	11
WTB-G8-1	+	3	34.3 (0.2)	5
WTB-G8-2	+	3	34.3 (0.0)	5
WTB-G8-3	+	2	34.8 (0.4)	4
WTB-G8-4	+	1	36.7 (0.7)	1
WTB-G8-5	+	1	36.1 (0.4)	2
WTB-G8-6	+	0	37.4 (0.8)	1
WTB-G8-7	+	1	35.7 (0.0)	2
WTB-G8-8	+	0	36.8 (0.8)	1
WTB-G8-9	+	0	37.8 (ND)^d^	1
WTB-G8-10	+	2	34.0 (0.1)	7
***Gremmeniella abietina* (EU race)**				
64667	-	7,370	None	-
64668	-	5,509	None	-
64672	-	1,820	None	-
64673	-	9,569	None	-
65097	+	5912	25.4 (0.3)	1,608
65171	+	65,081	21.9 (0.2)	19,176
65181	-	916	None	-
65539	-	26,396	None	-
67161	+	16,236	24.1 (0.0)	4,139
***Rosellinia necatrix***				
A	+	15,891	24.6 (0.1)	340
B	+	2,064	25.1 (0.1)	250
D	+	5,842	28.8 (0.0)	25
E	+	3,974	26.9 (0.1)	84
H	+	24,804	25.9 (0.0)	153
I	+	3,091	25.4 (0.2)	211
***Sclerotinia pseudotuberosa***				
1C	+	6,492,200	21.3 (0.1)	23,180
2C	+	5,530,494	21.6 (0.1)	18,600
***Phytophthora ramorum***				
16883	+	12,377	33.6 (0.2)	< 1
16885	+	17,646	33.2 (0.2)	3
17085	+	3,883	39.1 (0.2)	<1
17385	+	10,515	35.8 (0.1)	1
17358	+	10,131	37.4 (0.0)	< 1
07-Qr3-2i	+	10	None	-
07-Ab3-1i	+	4	34.2 (0.3)	2
07-As2-4i	+	44	38.3 (0.7)	< 1
07-Ll1-3i	+	15	34.6 (0.2)	1
07-Fa3-1i	+	686	28.2 (0.1)	63
07-Ba1-2i	+	2,254	26.5 (0.1)	170
07-Rho1-4i	+	2,743	26.0 (0.4)	241
07-Rho1-2c	-	0	None	-
02045	-	0	None	-
19347	-	9	None	-
20181	-	263	None	-
20644	-	10	None	-
20816	-	758	None	-
***Phytophthora kernoviae***				
16833	+	7,911	30.5 (0.4)	570
16876	+	983	31.7 (0.2)	292
17072	+	76	35.0 (0.4)	47
02045	-	0	None	-
19347	-	9	None	-
20644	-	10	None	-

^a^ According to the environmental samples’ provider.

^b^ Calculated with the results obtained from a SYBRGreen real-time PCR reaction.

^c^ Values obtained by plotting Ct values from the species-specific TaqMan assay into the standard curve-derived equation (Fig 2).

^d^ ND: one of the two replicates did not amplify.

One of the most critical steps when dealing with environmental samples is the quality of the DNA sample, which may vary with the extraction protocol used. In spite of this, detection limits as low as one target gene region copy were obtained (Table 6). For some samples, we obtained a positive result, i.e. a detectable Ct value using the TaqMan specific assays, but the calculated target gene region copy number was less than one. These results might be explained by the fact that we used standard curve equations to extrapolate those copy number values. Therefore, there exists a certain level of imprecision that has a more important effect on samples with a low level of target pathogen DNA. This imprecision caused by the extrapolation of target gene region copy values can also explain why, in some other cases (e.g. *C*. *fagacearum*, *G*. *morbida*, *F*. *circinatum* and *S*. *pseudotuberosa*), we obtained a slightly higher value for the target gene region than the one obtained with the genus assay (Table 6). The opposite result was also seen; for some environmental samples (e.g. *C*. *laricicola*, *C*. *polonica*, *G*. *abietina* (EU race), *R*. *necatrix*, *S*. *pseudotuberosa* and *P*. *ramorum*), the genus gene copy number was much higher than the target gene region copy number. This is most probably due to the presence of more than one species from the targeted genus in the environmental samples. For example, *G*. *abietina* (EU race) positive environmental samples were obtained from infected *Pinus resinosa* samples from the province of Québec, Canada. We know that the North American race of *G*. *abietina*, which is detected and counted with the genus assay but not with the EU race specific assay, might also be present on red pines [41]. Other examples are *Rhododendon* sp., *Griselinia* sp., and *Magnolia* x *soulangeana* plant tissues infected with *P*. *ramorum*. Those plants are well known to be hosts for other *Phytophthora* species [42, 43], which might explain the high level of quantification at the genus level, concomitant with a low level of *P*. *ramorum* itself.

Inhibitors from plant material or insect specimens co-extracted with DNA are a source of contamination that can impact PCR amplification accuracy [44–46], which is the variation between observed and expected data [24]. This property was evaluated for some of those assays (*C*. *polonica* and *C*. *laricola*) in a previous study [47]. In those specific cases, the presence of environmental DNA or any other co-extracted compound had no effect on the performance of the TaqMan assays. However, we are aware that assay performance could vary slightly depending on the material it is tested against. In fact, inhibition may be caused by the presence of different compounds such as acidic plant polysaccharides [48, 49], plant phenolics [50], the contamination of DNA samples with co-extracted polyphenol-bound proteins from the insect cuticle [51], or with phenolics and tannins found in the digestive tracts of xylophagous insects [45]. Accuracy evaluation should therefore be one of the initial steps for any user of those assays dealing with new environmental material.

We developed sensitive and specific molecular assays for ten alien tree pathogens identified as high priority potential threats for Canadian forests: *Ceratocystis fagacearum*, *Ceratocystis laricicola*, *Ceratocystis polonica*, *Fusarium circinatum*, *Gremmeniella abietina* (EU race), *Geosmithia morbida*, *Phytophthora kernoviae*, *Phytophthora ramorum*, *Rosellinia necatrix* and *Sclerotinia pseudotuberosa*. All of these assays are specific, i.e. they have the ability to amplify a unique DNA fragment of interest without amplifying or detecting non-target sequences. Detection assays were already available for some of the target tree pathogens selected. In some cases, they were included in our tree pathogen TaqMan assay panel (e.g. *C*. *polonica* and *C*. *laricicola* [47]). However, in most cases, existing assays had to be redesigned either because 1) they were not compliant with our real-time PCR conditions (*C*. *fagacearum* [52], *F*. *circinatum* [40, 53], *P*. *kernoviae* [54], *P*. *ramorum* [9, 54–59], *S*. *pseudotuberosa* [60]), 2) they were not tested against all closely related species (*F*. *circinatum* [61], *P*. *kernoviae* [7, 62], *P*. *ramorum* [7, 55, 63–65], *R*. *necatrix* [66–68]), or 3) they did not target the specific race of interest (*G*. *abietina* EU race [69]).

All assays were designed to be used under the same real-time PCR conditions, using the same chemistry and the same thermocycling parameters. Therefore, they can be performed in micro-well plates arrayed in any machine format to suit individual users’ needs and to increase throughput. Reactions for multiple samples, targeting multiple pathogens, can be performed in a single real-time PCR run, which is an important advantage under operational conditions where testing a large number of samples against of large number of targets is required. Molecular detection of these pathogenic species directly from plant material or insect vectors represents a powerful tool to prevent their introduction and establishment as potential invasive species.

## Supporting Information

S1 File Identification of unique gene models to *Phytophthora kernoviae* and *Phytophthora ramorum*. (DOCX)
