# Supplementary material for: Molecular Detection of 10 of the Most Unwanted Alien Forest Pathogens in Canada Using Real-Time PCR
Source: PLoS One. 2015 Aug 14;10(8):e0134265. doi: 10.1371/journal.pone.0134265 (PMC4537292; doi:10.1371/journal.pone.0134265)
Supplement: S1 File — (DOCX) [file pone.0134265.s001.docx]

**Identification of unique gene models to *Phytophthora kernoviae* and *Phytophthora ramorum***

The genome of the *Phytophthora kernoviae* isolate CBS 122049 was obtained by pair-end Illumina sequencing at Canada's Michael Smith Genome Sciences Centre (Vancouver, Canada). Two genomic DNA libraries with fragment size ranges of approximately 250 and 800 bp were constructed, according to British Columbia Cancer Agency Genome Sciences Centre tube-based and paired-end library protocols. Briefly, 1 µg of high molecular weight genomic DNA was sonicated (Covaris E210, Covaric Inc., Woburn, MA, USA) in 60 µl volume to 200-300 and ~800 bp fragments. Sonicated DNA was purified with Agencourt AMPure XP magnetic beads (Beckman Coulter, Mississauga, ON, Canada). The DNA fragments were end-repaired, phosphorylated and bead purified in preparation for A-tailing.  Illumina sequencing adapters were ligated overnight at 16^°^C and adapter ligated products were bead purified and enriched with 10 cycles of PCR using primers containing a hexamer index that enables library pooling. Paired-end 100 base reads were sequenced per pool in a single lane of an Illumina HiSeq 2000 instrument (Illumina Inc., San Diego, CA, USA).

Raw fastq reads were filtered by trimming the barcode sequence and poor quality reads using the PRINSEQ lite 0.20.4 [[1](#_ENREF_1)]. Trimmed reads were assembled using ABySS version 1.3.5 [[2](#_ENREF_2)] at four different k-mer values (k=36, 44, 54, 68). The assembly at k54 was selected and contigs below 500 bp were discarded, resulting in a 39.6 Mb assembly of 13,710 contigs with a N50 of 4,924. The CEGMA comparative framework [[3](#_ENREF_3)] was then used to estimate the completeness of the gene space in the assembled genome. Gene and protein model predictions and annotations were obtained by using AUGUSTUS [[4](#_ENREF_4)] trained with models from *Phytophthora sojae*, *Phytophthora infestans* and *Phytophthora ramorum* available on the JGI server [[5](#_ENREF_5)].

The 10,012 protein models predicted for *P. kernoviae* were combined with protein models from the sequenced genomes of *P. infestans* (18,179 models) [[6](#_ENREF_6)], *P. sojae* (26,584 models) [[7](#_ENREF_7)], *P. ramorum* (15,743 models) [[7](#_ENREF_7)], *Phytophthora capsicii* (19,805 models) [[8](#_ENREF_8)] and *Phytophthora cinnamomi* var. *cinnamomi* (26,131 models) [[5](#_ENREF_5)] and clustered with the orthoMCL algorithm (blast, inflation parameter of 1.5) [[9](#_ENREF_9)]. The 830 putative unique protein models to *P. kernoviae* identified in the clustering analysis were then submitted to a false positive filtering by running a TBLASTn (1e-20) on the *Phytophthora* genomes included in the study and a BLASTp (1e-20) on NCBI-nr. Positive hits were discarded and the gene sequence was retrieved for each of the 55 protein models left before being submitted to Primer 3 version 4.0.0 [[10](#_ENREF_10)] for PCR primer and probes identifications with the following parameters: product size of 100 to 150 bp, primers optimal size of 20 bp (18 to 27 bp) with an optimal Tm of 60°C (58 to 62°C); probe optimal size of 20 bp (18 to 27 bp) with an optimal Tm of 65°C (64 to 68°C).

The same process was applied to identify unique protein model for *P. ramorum*, expected the proteome/genomes of *Phytophthora lateralis* (17,533 models)*, Phytophthora hibernalis* (20,209 models) and *Phytophthora foliorum* (15,849 models) [Tyler *et al.,* personal communication] were added into the orthoMCL clustering analysis. After the false positive filtering stage with TBLASTn and BLASTp (see above), the search provided 30 unique protein models to *P. ramorum*.

**References**

1. Schmieder R, Edwards R (2011) Quality control and preprocessing of metagenomic datasets. Bioinformatics 27: 863-864.

2. Simpson JT, Wong K, Jackman SD, Schein JE, Jones SJM, et al. (2009) ABySS: A parallel assembler for short read sequence data. Genome Research 19: 1117-1123.

3. Parra G, Bradnam K, Ning Z, Keane T, Korf I (2009) Assessing the gene space in draft genomes. Nucleic Acids Research 37: 289-297.

4. Keller O, Kollmar M, Stanke M, Waack S (2011) A novel hybrid gene prediction method employing protein multiple sequence alignments. Bioinformatics 27: 757-763.

5. Grigoriev IV, Nordberg H, Shabalov I, Aerts A, Cantor M, et al. (2011) The Genome Portal of the Department of Energy Joint Genome Institute. Nucleic Acids Research.

6. Haas BJ, Kamoun S, Zody MC, Jiang RHY, Handsaker RE, et al. (2009) Genome sequence and analysis of the Irish potato famine pathogen *Phytophthora infestans*. Nature 461: 393-398.

7. Tyler BM, Tripathy S, Zhang X, Dehal P, Jiang RHY, et al. (2006) *Phytophthora* genome sequences uncover evolutionary origins and mechanisms of pathogenesis. Science 313: 1261-1266.

8. Lamour KH, Mudge J, Gobena D, Hurtado-Gonzales OP, Schmutz J, et al. (2012) Genome sequencing and mapping reveal loss of heterozygosity as a mechanism for rapid adaptation in the vegetable pathogen *Phytophthora capsici*. Molecular Plant-Microbe Interactions 25: 1350-1360.

9. Li L, Stoeckert CJ, Roos DS (2003) OrthoMCL: Identification of ortholog groups for eukaryotic genomes. Genome Research 13: 2178-2189.

10. Untergasser A, Cutcutache I, Koressaar T, Ye J, Faircloth BC, et al. (2012) Primer3—new capabilities and interfaces. Nucleic Acids Research 40: e115.
